# SOX12 promotes colorectal cancer cell proliferation and metastasis by regulating asparagine synthesis

**DOI:** 10.1038/s41419-019-1481-9

**Published:** 2019-03-11

**Authors:** Feng Du, Jie Chen, Hao Liu, Yanhui Cai, Tianyu Cao, Weili Han, Xiaofang Yi, Meirui Qian, Dean Tian, Yongzhan Nie, Kaichun Wu, Daiming Fan, Limin Xia

**Affiliations:** 1 State Key Laboratory of Cancer Biology, National Clinical Research Center for Digestive Diseases and Xijing Hospital of Digestive Diseases, Fourth Military Medical University, Xi’an, 710032 Shaanxi Province China; 2 Department of Psychiatry, Xijing Hospital, Fourth Military Medical University, Xi’an, 710032 China; 3 Department of Gastroenterology, Tongji Hospital of Tongji Medical College, Huazhong University of Science and Technology, Wuhan, 430030 Hubei Province China

## Abstract

The sex-determining region Y (SRY)-box (SOX) family has a crucial role in carcinogenesis and cancer progression. However, the role of SOX12 and the mechanism by which it is dysregulated in colorectal cancer (CRC) remain unclear. Here we analyzed SOX12 expression patterns in two independent CRC cohorts (cohort I, *n* = 390; cohort II, *n* = 363) and found that SOX12 was significantly upregulated in CRC, indicating a poor prognosis in CRC patients. Overexpression of SOX12 promoted CRC cell proliferation and metastasis, whereas downregulation of SOX12 hampered CRC aggressiveness. Mechanistically, SOX12 facilitated asparagine synthesis by transactivating glutaminase (*GLS*), glutamic oxaloacetic transaminase 2 (*GOT2*), and asparagine synthetase (*ASNS*). Downregulation of GLS, GOT2, and ASNS blocked SOX12-mediated CRC cell proliferation and metastasis, whereas ectopic expression of GLS, GOT2, and ASNS attenuated the SOX12 knockdown-induced suppression of CRC progression. In addition, serial deletion, site-directed mutagenesis, luciferase reporter, and chromatin immunoprecipitation (ChIP) assays indicated that hypoxia-inducible factor 1α (HIF-1α) directly binds to the SOX12 promoter and induces SOX12 expression. Administration of l-asparaginase decreased SOX12-mediated tumor growth and metastasis. In human CRC samples, SOX12 expression positively correlated with GLS, GOT2, ASNS, and HIF-1α expression. Based on these results, SOX12 may serve as a prognostic biomarker and l-asparaginase represents a potential novel therapeutic agent for CRC.

## Introduction

Colorectal cancer (CRC) is the second most common cancer in females and the third most common cancer in males^1^. Metastasis and recurrence are two of the major causes of CRC-related death^2^. With rapid increases in genetic data and the development of new technologies, numerous advances have been achieved in the diagnosis and treatment of CRC^3^. However, the molecular mechanisms underlying CRC initiation and progression, which are urgently needed for the diagnosis and treatment of CRC, remain unclear.

**Fig. 1:**
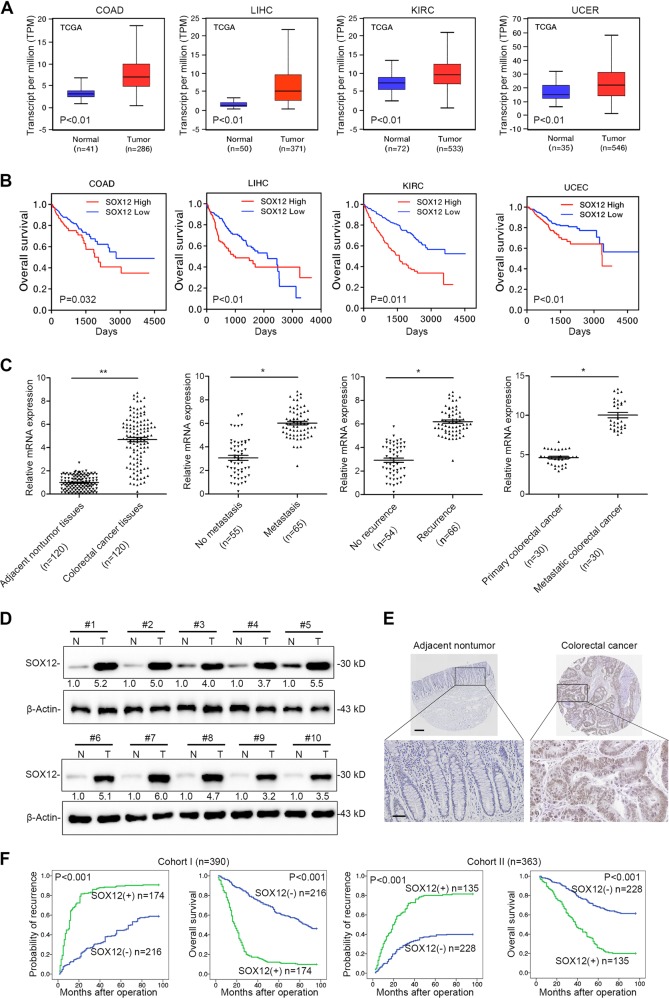
Elevated expression of SOX12 indicates a poor prognosis in patients with CRC. **a** Representative data extracted from TCGA datasets showing the relative expression of SOX12 mRNA in multiple cancer tissues compared with normal tissues. The box-and-whisker plots show the medians (horizontal lines), interquartile ranges (boxes), and minimum and maximum values (whiskers) of the data. COAD colon adenocarcinoma, KIRC kidney renal clear cell carcinoma, LIHC liver hepatocellular carcinoma, UCEC uterine corpus endometrial carcinoma; **P < 0.01 and *P < 0.05. **b** Kaplan–Meier analysis showing the correlation between SOX12 mRNA expression and OS for the patients included in the TCGA datasets. **c** RT-PCR analysis showing the pattern of SOX12 expression in human CRC tissues compared with adjacent nontumor tissues, metastatic CRC compared with nonmetastatic CRC, recurrent CRC compared with nonrecurrent CRC, and metastatic CRC compared with primary CRC. **d** Western blotting analyses performed using human CRC tissues and adjacent nontumor tissues. **e** IHC staining for SOX12 in human CRC tissues. The scale bars represent 200 µm (upper panel) and 50 µm (lower panel). **f** Kaplan–Meier analysis revealed a positive correlation between SOX12 expression and tumor recurrence, and a negative correlation with OS. *P < 0.05, **P < 0.01 compared with the control. The data are presented as the mean ± SD

The sex-determining region Y-associated high-mobility group (HMG) cassette (SOX) transcription factor family currently comprises 20 members in most vertebrates and is characterized by the DNA-binding HMG-box domain^4,5^. Based on sequence homology and other structural motifs within the HMG domain, the SOX family is subdivided into eight groups, A–H. All SOX genes display specific expression patterns and have key roles in stem cell maintenance and cell-fate determination during development^5,6^. Among the groups of SOX family proteins, group C includes three proteins present in most vertebrates: SOX4, SOX11, and SOX12^6,7^; these proteins have been well studied in cancer and in important developmental processes^8–12^. For example, SOX4 promotes cancer metastasis by inducing the epithelial–mesenchymal transition in acute myeloid leukemia, breast cancer, and prostate cancer^9,10,13^. SOX11 is found as a double-edged sword in mantle cell lymphoma; it promotes tumor angiogenesis to facilitate cancer cell proliferation and metastasis^14^, but patients with low SOX11 expression have shorter overall survival (OS) times^15^. Interestingly, SOX12 has been reported to promote tumor progression^6,16,17^ and datasets from the Cancer Genome Atlas (TCGA) indicate that SOX12 upregulation is correlated with poor prognosis in multiple types of cancer, including CRC (Fig. 1a, b). However, according to Duquet et al.^18^, SOX12 knockdown in CRC cells HT29 enhances metastasis. These conflicting findings have prompted a need for further investigations aimed at exploring the ambiguous role of SOX12 in CRC.

Cancer cells present a wide array of metabolic abnormalities^19,20^. Such reprogramming is now recognized as a hallmark of cancer^19,20^. Examining metabolic abnormalities provides a new therapeutic perspective to improve the diagnosis and treatment of cancer^21,22^. Recently, a study screening biomarkers of metabolic reprogramming in mouse and human colorectal tumors reported significant increases in amino acid metabolites, including glutamic acid, proline, and arginine, implying an important role of glutamine–asparagine disturbances in CRC^23^. Notably, aspartate was recently reported as an endogenous metabolite limiting tumor growth^24^. Asparagine bioavailability drives breast cancer metastasis and protects tumor cells from glutamine depletion-induced cell death^25^. Based on these findings, asparagine biosynthesis has a key role in accelerating cancer growth and metastasis. Moreover, the enzyme l-asparaginase has been used to treat lymphoblastic malignancies in children^26^. In non-small cell lung cancer, the proto-oncogene Kirsten rat sarcoma (KRAS) controls asparagine biosynthesis via ATF4 and changes the sensitivity of l-asparaginase. These findings have led to the concept that l-asparaginase is a viable therapeutic molecule for malignant diseases^27^. Nonetheless, the role of asparagine in CRC and the mechanism underlying asparagine dysregulation remain to be elucidated.

In this study, we report for the first time the role and clinical significance of SOX12 in CRC. SOX12 overexpression is correlated with CRC metastasis and suggests a poor prognosis. SOX12, a direct target of hypoxia-inducible factor 1α (HIF-1α), promotes CRC cell proliferation and metastasis by regulating three key enzymes, glutaminase (GLS), glutamic oxaloacetic transaminase 2 (GOT2), and asparagine synthetase (ASNS), in asparagine biosynthesis. Moreover, administration of l-asparaginase suppresses SOX12-mediated CRC cell proliferation and metastasis.

## Results

### Elevated expression of SOX12 indicates a poor prognosis for patients with CRC

We first investigated the expression of SOX12 in TCGA datasets and observed significantly higher SOX12 expression in multiple cancer specimens than in normal tissues (Fig. 1a and Supplementary Figure S1). Furthermore, Kaplan–Meier analysis based on TCGA datasets revealed that patients with high levels of SOX12 had shorter OS than patients with low levels of SOX12 (Fig. 1b), strongly suggesting that SOX12 contributes to the progression of these human cancers. In this study, we were particularly interested in identifying the role of SOX12 in CRC. To this end, expression of SOX12 was analyzed in 120 pairs of CRC tissues and corresponding adjacent tissues using quantitative reverse transcription PCR (qRT-PCR). SOX12 was significantly upregulated in CRC tissues compared with adjacent normal tissues and was expressed at higher levels in CRC tissues from patients with metastases (65 of 120) than in those from nonmetastatic patients (55 of 120, Fig. 1c). Patients with CRC recurrence (66 of 120) exhibited higher expression of SOX12 mRNA than did patients without CRC recurrence (54 of 120). In addition, SOX12 expression were increased in metastatic CRC tissues compared with the paired primary CRC tissues (*n* = 30) (Fig. 1c). Consistent with the mRNA levels, the SOX12 protein levels were also significantly higher in CRC tissues than in adjacent nontumor tissues (Fig. 1d, e).

Furthermore, SOX12 expression and its clinical significance were analyzed in a cohort of CRC tissues (*n* = 390, cohort I) using immunohistochemical (IHC) staining. SOX12 overexpression was significantly associated with tumor invasion, tumor differentiation, lymph node metastasis, distant metastasis, and American Joint Committee on Cancer (AJCC) staging (Supplementary Table S1), and was found to be an independent risk factor for reduced survival and recurrence in patients (Supplementary Table S2). Patients with higher SOX12 levels had shorter OS times and higher relapse rates than did patients with lower SOX12 expression (Fig. 1f). Similar findings were confirmed in an independent cohort of CRC tissues (cohort II; *n* = 363). SOX12 upregulation was significantly associated with high invasiveness (Supplementary Table S1) and poor prognosis (Fig. 1f). High SOX12 expression was a predictor of OS and postoperative recurrence (Supplementary Table S3). Taken together, these findings suggested that SOX12 was upregulated in CRC and indicated a poor prognosis.

### SOX12 promotes CRC cell proliferation, migration, and invasion in vitro

**Fig. 2:**
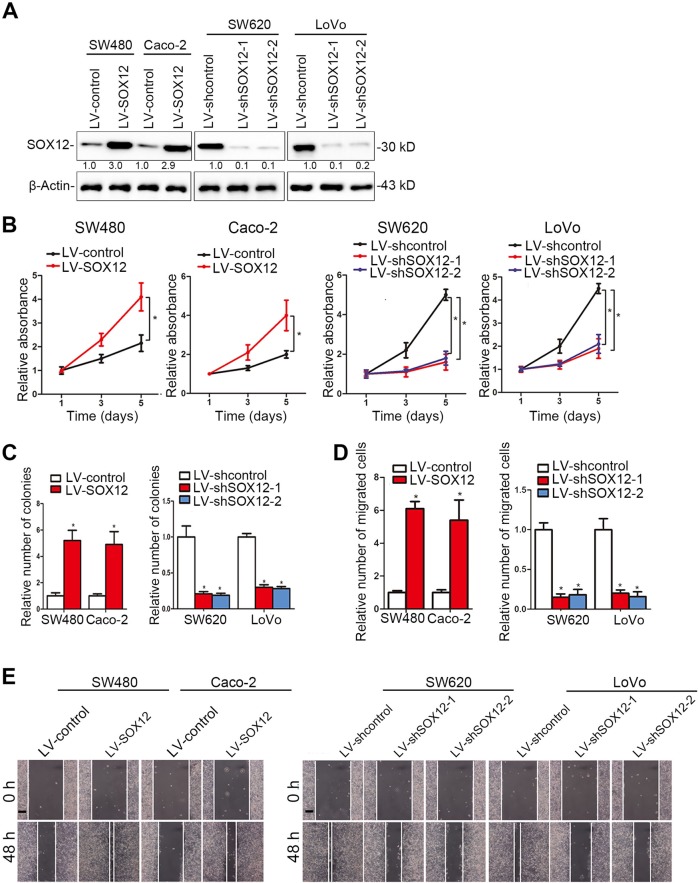
SOX12 promotes CRC cell proliferation, migration and invasion in vitro. **a** Western blottings showing the effects of lentiviral infection of the indicated human CRC cells. **b** The effects of SOX12 on CRC cell proliferation were measured using a CCK-8 assay. **c** Effects of SOX12 on human CRC cell colony formation. **d** Transwell assays using the indicated cells. **e** Effects of SOX12 on wound-healing in the indicated cells. The scale bar represents 100 µm. *P < 0.05 compared with the control. The data are presented as the mean ± SD

We examined the levels of SOX12 in CRC cell lines and found that SOX12 was expressed at much higher levels in CRC cell lines with high metastatic potential than in CRC cell lines with low metastatic potential (Supplementary Figure S2). Then, we established four stable cell lines to clarify the role of SOX12 in the progression of CRC (Fig. 2a). Cell Counting Kit 8 (CCK-8) assays indicated that SOX12 overexpression significantly increased CRC cell proliferation (Fig. 2b), which was consistent with the results of the colony formation assay (Fig. 2c). However, SOX12 knockdown suppressed the proliferation and colony formation capability of CRC cells (Fig. 2b, c). In addition, SOX12 overexpression increased the invasion and migration of SW480 and Caco-2 cells, whereas SOX12 knockdown reduced the invasion and migration of SW620 and LoVo cells (Fig. 2d, e).

To eliminate the impacts of other genetic differences between the cell lines, we further upregulated and downregulated SOX12 expression in the same cell lines (SW480 and Caco-2) (Supplementary Figure S3A). Consistent with our previous results, SOX12 overexpression promoted SW480 and Caco-2 cell proliferation, colony formation, migration, and invasion. In contrast, SOX12 knockdown in SW480 and Caco-2 cells inhibited their malignant phenotypes (Supplementary Figure S3B-D). Taken together, these findings suggest that SOX12 functions as an oncogene to promote CRC cell proliferation, migration, and invasion in vitro.

### SOX12 promotes CRC cell tumorigenesis and metastasis in vivo

**Fig. 3:**
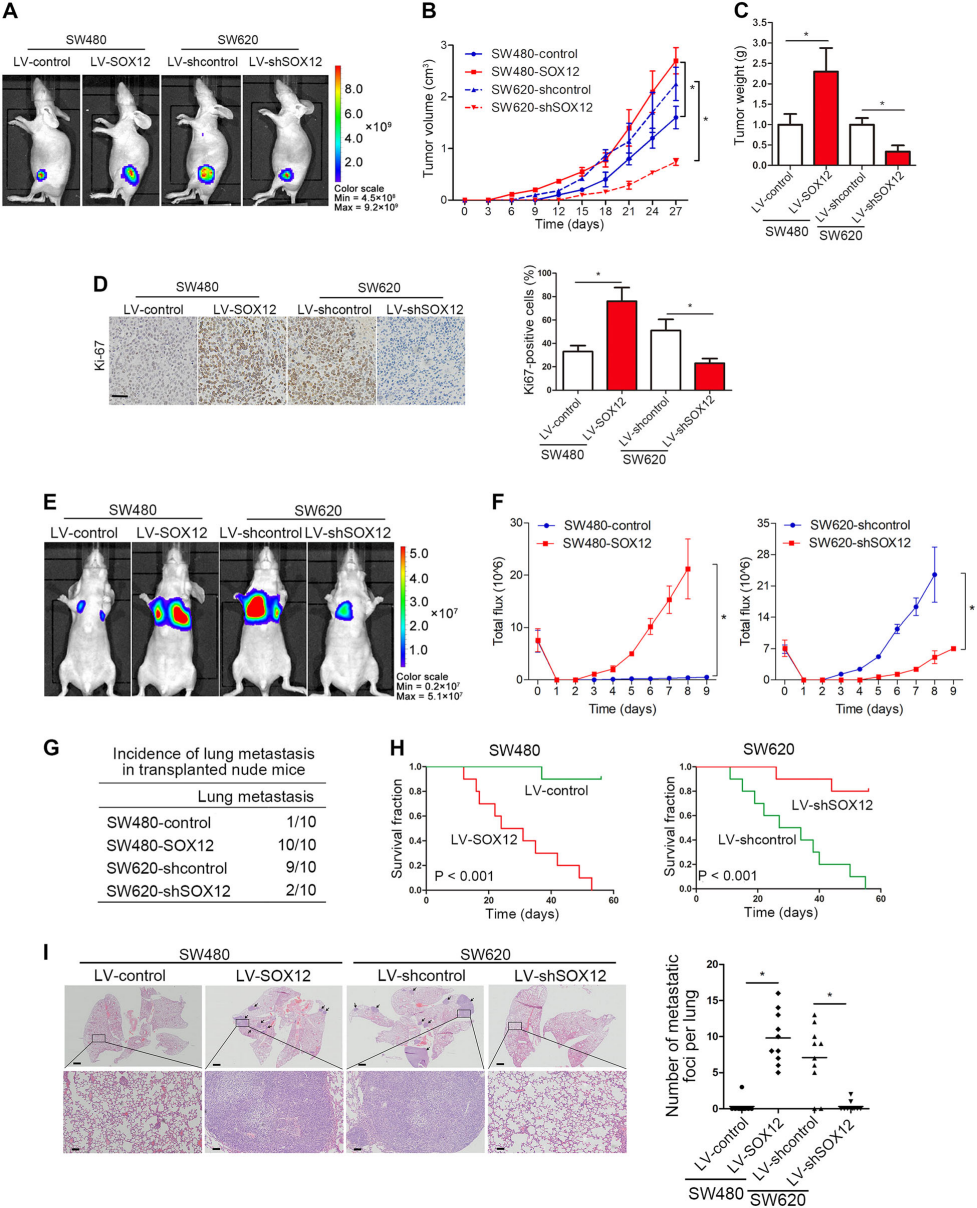
SOX12 promotes CRC cell tumorigenesis and metastasis in vivo. **a** The indicated cells were subcutaneously injected into nude mice (*n* = 10 mice per group). Representative fluorescence images of luciferase signals captured from subcutaneous tumors are shown. **b** Growth curves of tumors in nude mice (*n* = 10 mice per group) injected with the indicated cells. **c** Tumors (*n* = 10) were isolated and weighed 28 days after the injection. **d** IHC staining for Ki67 and the percentage of Ki67-positive cells in the indicated tumors. The scale bars represent 50 µm. **e** Representative BLI scans of lung metastasis in the different groups at 9 weeks after implantation. **f** The bioluminescence intensity at the indicated time points is presented as the total photon flux. **g** Incidence of lung metastasis in the indicated groups of nude mice. **h** Overall survival time in the different groups of nude mice. **i** Representative images of H&E-stained lung tissues from the different groups. The scale bars represent 200 µm (upper panel) and 50 µm (lower panel). The number of lung metastatic nodules is shown. *P < 0.05 compared with the control. The data are presented as the mean ± SD

A subcutaneous xenograft nude mice model was established for tumorigenesis and the results showed that SOX12 overexpression significantly increased tumor growth, whereas SOX12 inhibition suppressed tumor growth (Fig. 3a–c). IHC staining for Ki67 in the xenografts revealed significant increases in the proliferation of tumor cells overexpressing SOX12 (Fig. 3d). Moreover, in vivo lung metastasis experiments showed that ectopic SOX12 expression significantly increased and SOX12 knockdown reduced CRC lung metastasis, as measured from the bioluminescence signals (Fig. 3e, f). SOX12 overexpression increased lung metastasis incidence and shortened the OS of mice in the SW480-SOX12 group, whereas SOX12 knockdown reduced the lung metastasis rate and increased the OS of mice in the SW620-shSOX12 group (Fig. 3g, h). Hematoxylin and eosin (H&E) staining revealed an increased number of lung metastatic nodules upon SOX12 overexpression, whereas SOX12 knockdown produced the opposite effect (Fig. 3i). Based on these findings, SOX12 functions as an oncogene that promotes CRC cell tumorigenesis and metastasis in vivo.

### SOX12 regulates asparagine synthesis by transactivating *GLS*, *GOT2*, and *ASNS* expression in human CRC cells

**Fig. 4:**
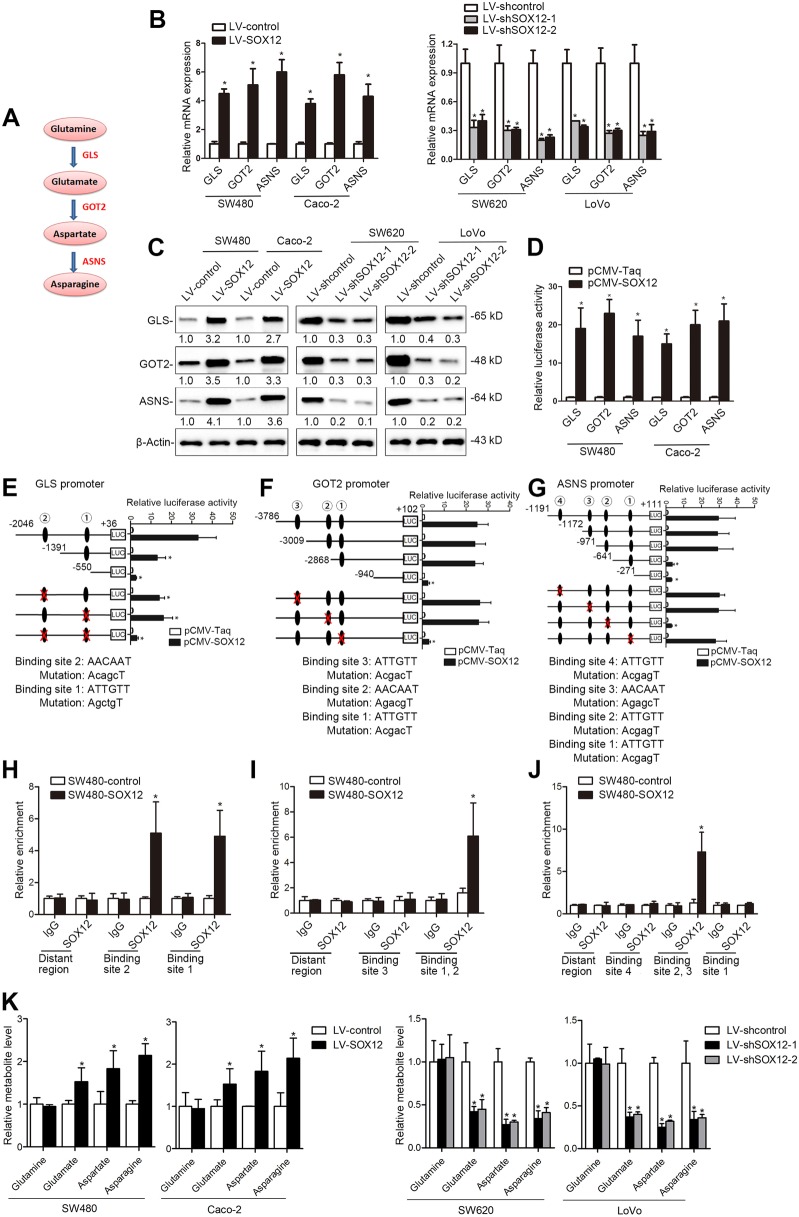
SOX12 regulates asparagine synthesis by transactivating *ASNS*, *GLS*, and *GOT2* expression in human CRC. **a** ASNS, GLS, and GOT2 are three key enzymes in asparagine synthesis. **b**, **c** After CRC cells were infected with LV-SOX12 or LV-shSOX12, GLS, GOT2, and ASNS levels were detected using qRT-PCR (**b**) and western blotting (**c**, **d**). After cotransfection of the luciferase constructs containing the (− 2046/ + 36) GLS, (− 3786/ + 102) GOT2, or (− 1191/ + 111) ASNS promoters with pCMV-SOX12, the relative luciferase activity was determined. **e**–g) Serially truncated and mutated GLS (**e**), GOT2 (**f**), and ASNS (**g**) promoter plasmids were cotransfected with pCMV-SOX12, and promoter luciferase assays were performed. **h**–**j** A ChIP assay revealed direct interactions between SOX12 and the GLS (**h**), GOT2 (**i**), and ASNS (**j**) promoters in CRC cells. **k** The levels of the indicated intracellular metabolites in SW480, Caco-2, SW620, and LoVo cells were analyzed using LC-MS/MS. *P < 0.05 compared with the control. The data are presented as the mean ± SD

Tumor cells experience fluctuating amino acid availability^28,29^. Given that amino acids are the building blocks of proteins and intermediate metabolites that activate other biosynthetic pathways^30,31^, we sought to determine whether SOX12 affects amino acid metabolism to promote CRC progression. To this end, we used an Amino Acid Metabolism RT^2^ Profiler PCR Array to examine transcriptome variations mediated by SOX12 overexpression in SW480 cells and to investigate whether SOX12 regulated amino acid metabolism to promote CRC progression. With a twofold change as the cutoff, 60 of the 168 amino acid metabolism-related genes were upregulated and 22 genes were downregulated in SW480 cells upon SOX12 overexpression; 86 genes showed no significant change (Supplementary Table S4). Among the upregulated genes, *ASNS*, *GLS*, and *GOT2* were strongly induced by SOX12 overexpression (Supplementary Table S4). *ASNS*, *GLS*, and *GOT2* encode ASNS, GLS, and GOT2, respectively, which are key enzymes in asparagine synthesis (Fig. 4a) and are all required for tumor growth and metastasis^32–34^, prompting the hypothesis that asparagine synthesis is required for SOX12-mediated CRC progression. Notably, GOT1 and GOT2 are the cytoplasmic and mitochondrial forms of glutamic oxaloacetic transaminase, respectively. SOX12 overexpression significantly increased GOT2 levels but did not change expression of GOT1 (Supplementary Table S4), suggesting that GOT2, rather than GOT1, is the major factor in SOX12-mediated asparagine synthesis.

We then detected the GLS, GOT2, and ASNS levels in CRC cells after SOX12 manipulation. SOX12 overexpression significantly increased the expression of GLS, GOT2, and ASNS, which was reduced by SOX12 knockdown (Fig. 4b, c). Luciferase reporter assays showed that SOX12 transactivated GLS, GOT2, and ASNS promoter activity (Fig. 4d). Through sequence analysis, we identified two SOX12-binding sites in the GLS promoter. The site-directed mutagenesis and serial deletion assays suggested that both SOX12-binding sites were critical for SOX12-induced GLS transactivation (Fig. 4e). Similarly, the GOT2 promoter region contains three possible SOX12-binding sites, but only the absence of the first SOX12-binding site disrupted the GOT2 transcription (Fig. 4f). In addition, serial fragment deletions and point mutations at four SOX12-binding sites in the ASNS promoter indicated that SOX12 transactivated *ASNS* expression by directly binding to the third SOX12-binding site of the ASNS promoter (Fig. 4g). Chromatin immunoprecipitation (ChIP) analyses further revealed enhanced binding of SOX12 to these regions in the *GLS, GOT2*, and *ASNS* promoters (Fig. 4h–j).

We applied targeted metabolomics using U-^13^C_5_-glutamine as a tracer to further elucidate the effect of SOX12 on asparagine synthesis. Liquid chromatography-tandem mass spectrometry (LC-MS/MS) analysis revealed significantly increased levels of metabolites in SW480 and Caco-2 cells overexpressing SOX12. In contrast, SOX12 knockdown in SW620 and LoVo cells clearly decreased the levels of glutamate, aspartate, and asparagine (Fig. 4k). Taken together, the results suggest that SOX12 is a master regulator of asparagine synthesis that acts by transactivating *GLS*, *GOT2*, and *ASNS*.

### GLS, GOT2, and ASNS are essential for SOX12-mediated CRC cell proliferation and metastasis

**Fig. 5:**
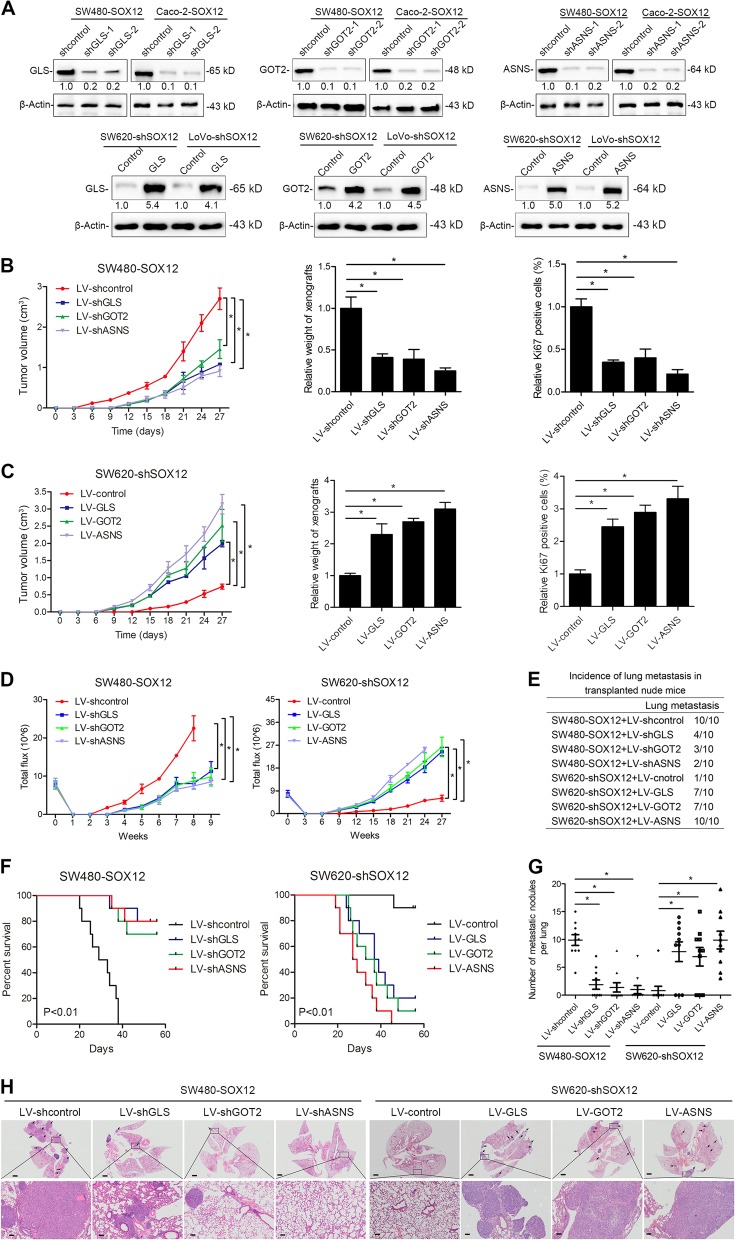
GLS, GOT2, and ASNS are essential for SOX12-mediated CRC cell proliferation and metastasis. **a** Western blotting analysis of GLS, GOT2, and ASNS levels in the indicated cells. **b** In vivo tumorigenesis assays showed that GLS, GOT2, and ASNS knockdown abolished SOX12-mediated tumorigenesis. The growth curves, tumor weights, and percentages of Ki67-positive cells in the indicated groups are shown. **c** In vivo tumorigenesis assays showed that GLS, GOT2, and ASNS overexpression attenuated the increase in CRC cell tumorigenesis induced by SOX12 knockdown. The growth curves, tumor weights, and percentages of Ki67-positive cells in the indicated groups are shown. **d** The BLI intensity of lung metastases in the different groups was traced for 9 weeks after implantation. The bioluminescence intensity at the indicated time points is presented as the total photon flux. **e** Incidence of lung metastasis in the indicated groups of nude mice. **f** Overall survival times in the indicated groups of nude mice. **g** Numbers of lung metastatic nodules. **h** Representative images of H&E-stained lung tissues from the different groups. The scale bars represent 200 µm (upper panel) and 50 µm (lower panel). *P < 0.05 compared with the control. The data are presented as the mean ± SD

We depleted GLS, GOT2, and ASNS in SOX12-overexpressing SW480 and Caco-2 cells, and upregulated GLS, GOT2, and ASNS in SOX12-knockdown SW620 and LoVo cells to verify the participation of GLS, GOT2, and ASNS in SOX12-mediated CRC cell proliferation and metastasis (Fig. 5a).

In vivo tumorigenesis experiments revealed significant decreases in tumor growth, tumor weight, and Ki67-positive staining upon silencing of GLS, GOT2, or ASNS in SW480-SOX12 cells (Fig. 5b), whereas GLS, GOT2, or ASNS overexpression reversed the SOX12 knockdown-induced suppression of tumor growth (Fig. 5c). In addition, GLS, GOT2, and ASNS silencing reduced bioluminescence signals, lung metastasis rates, and the numbers of lung metastatic nodules and increased the OS of mice in the SW480-SOX12 group (Fig. 5d–g). In contrast, GLS, GOT2, and ASNS overexpression increased the lung bioluminescence intensity, incidence of lung metastasis, and number of metastatic lung nodules, subsequently reducing the OS of mice in the SW620-shSOX12 group (Fig. 5d–g). Thus, GLS, GOT2, and ASNS are essential for SOX12-mediated CRC cell proliferation and metastasis.

### l-Asparaginase significantly inhibits CRC cell proliferation and metastasis

**Fig. 6:**
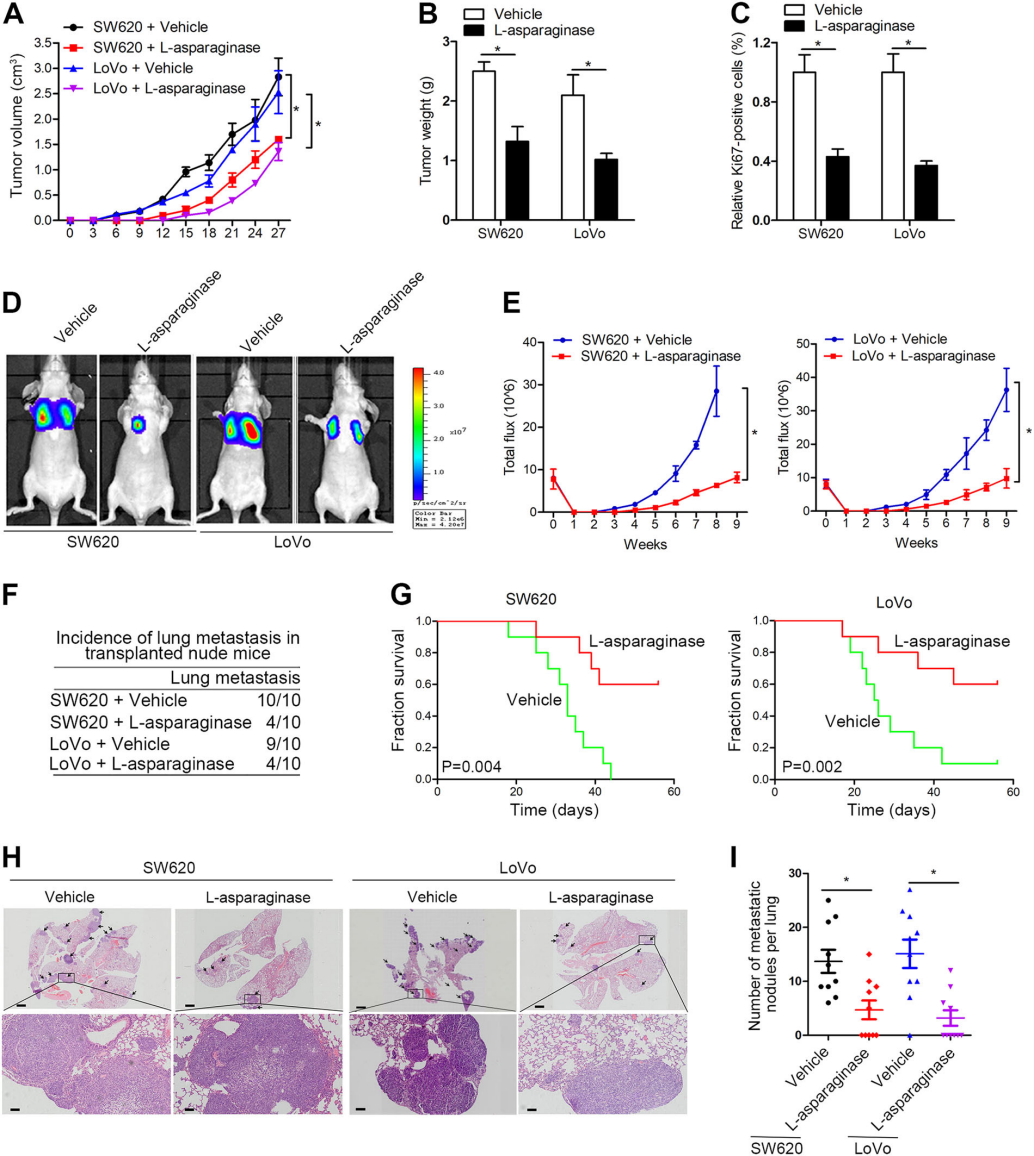
l-Asparaginase inhibits CRC cell tumorigenesis and metastasis. **a**–**c** Mice in different groups were intraperitoneally injected with saline control or l-asparaginase (2.0 mg/kg) daily when their tumors reached the determined size (approximately 100 mm^3^) (*n* = 10 mice per group). The tumors were isolated on day 28 after injection. Representative data for the tumor mass (**a**), volume (**b**), and weight (**c**) in the different groups are shown. **d**–**i** l-asparaginase inhibits CRC lung metastasis. **d** Representative BLI results indicating the metastasizing cells after 9 weeks. **e** The bioluminescence intensity in the cells at the indicated time points is presented as the total photon flux. **f** Incidence of lung metastasis in the transplanted mice. **g** Overall survival of the mice in each group. **h** Representative images of H&E-stained lung metastatic nodules. The scale bars represent 200 µm (upper panel) and 50 µm (lower panel). **i** Quantification of the tumor foci in the lungs of each group. *P < 0.05 compared with the control. The data are presented as the mean ± SD

As SOX12 promoted CRC progression through GLS/GOT2/ASNS-mediated asparagine synthesis, we sought to determine whether an asparagine inhibitor would be an effective treatment for CRC. An in vivo subcutaneous tumorigenesis model showed that l-asparaginase treatment significantly decreased tumor growth, tumor size, and Ki67-positive staining compared with vehicle treatment (Fig. 6a–c). Moreover, l-asparaginase reduced lung bioluminescence intensity, and the incidence of lung metastasis, whereas improving the OS of the l-asparaginase group compared with that of the control group (Fig. 6d–f). H&E staining showed a decrease in the number of lung metastatic nodules in transplanted nude mice treated with l-asparaginase (Fig. 6g). Based on these observations, l-asparaginase inhibits CRC progression and might be a promising treatment for CRC.

### SOX12 is transactivated by HIF-1α

**Fig. 7:**
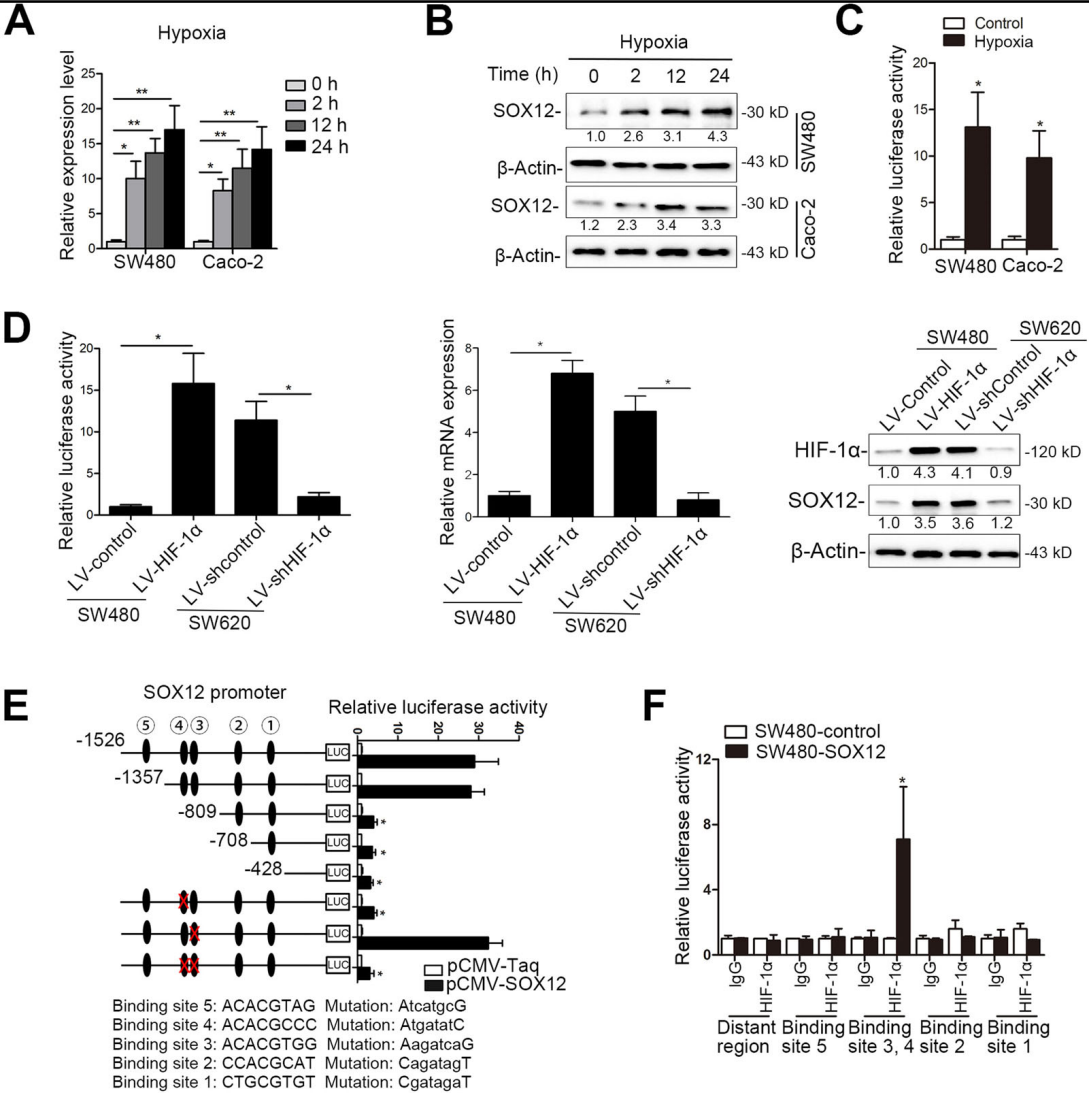
SOX12 is identified as a direct target gene of HIF-1α. **a**, **b** CRC cells were cultured under hypoxic (0.5% O_2_) conditions for the indicated time intervals and SOX12 expression was examined using qRT-PCR (**a**) and western blotting (**b**, **c**). A luciferase reporter construct carrying the (−1526/ + 150) SOX12 promoter was transfected into the indicated CRC cells, and luciferase activity was measured after 24 h. (**d**) SW480 cells and SW620 cells were separately infected with HIF-1α lentivirus (LV-HIF-1α) and shHIF-1α lentivirus (LV-shHIF-1α). SOX12 transcription and expression levels were measured after 24 h using luciferase assays (left panel), qRT-PCR (middle panel) and western blotting (right panel). (**e**) Truncated and mutated SOX12 promoter constructs were cotransfected with pCMV-HIF-1α, and the relative luciferase activity was confirmed. (**f**) A ChIP assay confirmed the direct binding of HIF-1α to the SOX12 promoter in CRC cells and human CRC tissues. *P < 0.05, **P < 0.01 compared with the control. The data are presented as the mean ± s.d

Hypoxia is a key driver in metabolic pathways that impact cell survival and drug resistance and contribute to recurrence and metastasis^35,36^. Recent studies have reported that hypoxia determines the regulation and utilization of amino acid metabolism in CRC cell lines, and that alanine, aspartate, and glutamate metabolism is significantly increased in hypoxic cells^37^. These findings suggest that hypoxia has key roles in regulating amino acid metabolism in human cancer cells. Interestingly, when we cultured SW480 and Caco-2 cells under hypoxic conditions (0.5% O_2_), we found that hypoxia significantly increased SOX12 expression in a time-dependent manner (Fig. 7a, b). CRC cells were transfected with a reporter plasmid containing the SOX12 gene promoter and subjected to hypoxia to examine whether hypoxia-induced SOX12 expression participated in the transactivation of its promoter. SOX12 transcription was significantly increased after hypoxia, as evidenced by increased luciferase activity (Fig. 7c). Overexpression of HIF-1α increased luciferase promoter activity and the levels of SOX12 mRNA and protein, whereas HIF-1α silencing exerted the opposite effects (Fig. 7d). We further analyzed the sequence of the SOX12 promoter to determine the potential *cis*-regulatory elements and identified five putative HIF-1α-binding sites. Deletions from nt-1357 and nt-809 significantly reduced SOX12 promoter activity. We also performed site-directed mutagenesis and found that mutation of the fourth HIF-1α-binding site significantly reduced hypoxia-induced SOX12 promoter activity (Fig. 7e). The ChIP assay results verified the direct binding of HIF-1α to the SOX12 promoter in CRC cells and human CRC tissues (Fig. 7f). Overall, these findings indicate that SOX12 is a direct target gene of HIF-1α.

### SOX12 expression positively correlates with GLS, GOT2, ASNS, and HIF-1α expression in CRC

**Fig. 8:**
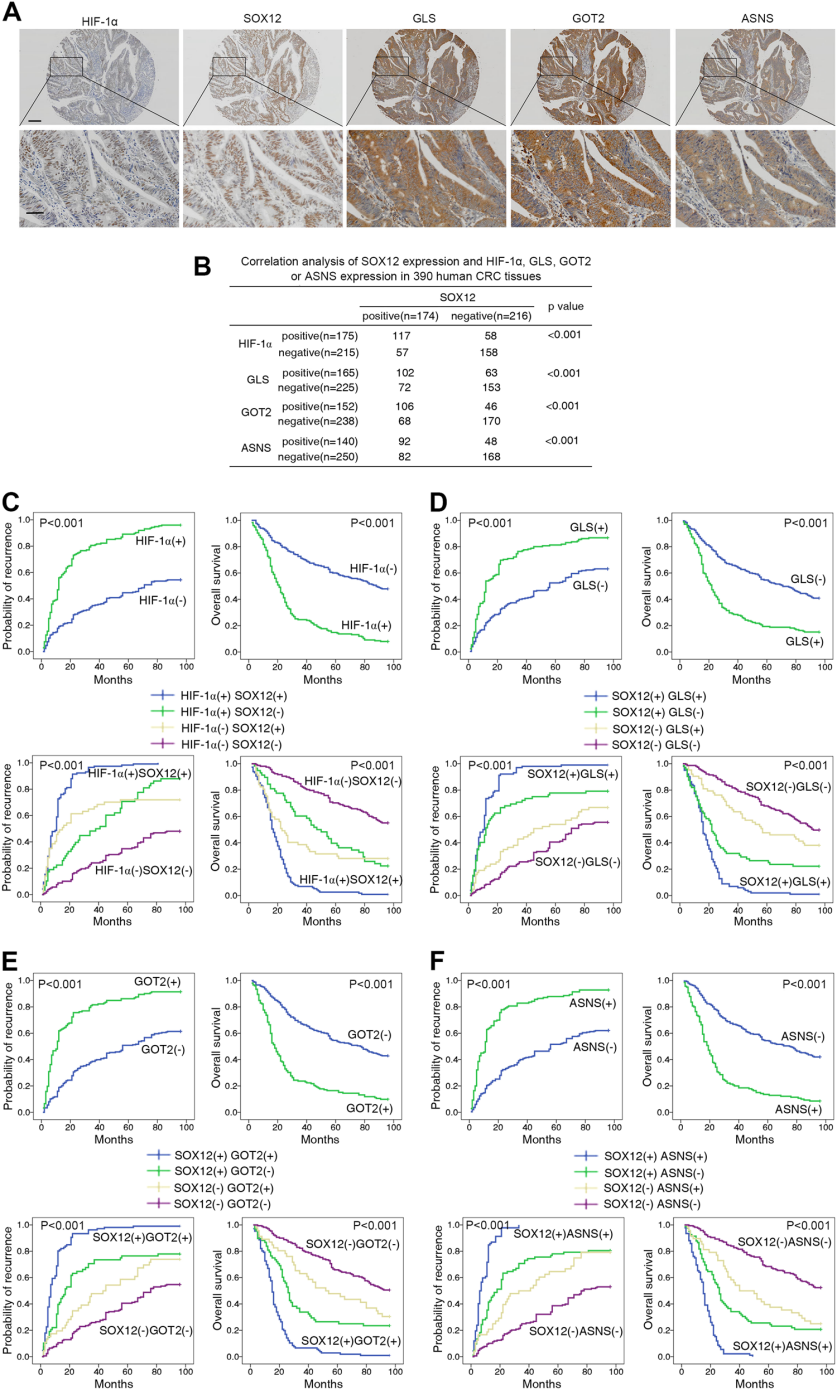
SOX12 expression positively correlates with GLS, GOT2, ASNS, and HIF-1α expression in CRC. **a** Representative images of IHC staining for SOX12, HIF-1α, GLS, GOT, and ASNS in human CRC tissues. **b** Analysis of correlations between SOX12 expression and GOT2 and ASNS expression in human CRC tissues. **c** Kaplan–Meier analysis of the associations between HIF-1α expression and the recurrence and overall survival of patients with CRC (upper panel). Kaplan–Meier analysis of the associations between concurrent SOX12 and HIF-1α expression and the recurrence and OS of patients with CRC (lower panel). **d** Kaplan–Meier analysis of the associations between GLS expression and the recurrence and OS of patients with CRC (upper panel). Kaplan–Meier analysis of the associations between simultaneous SOX12 and GLS expression and the relapse and OS of patients with CRC (lower panel). **e** Kaplan–Meier analysis of the associations between GOT2 expression and the relapse and OS of patients with CRC (upper panel). Kaplan–Meier analysis of the associations between simultaneous SOX12 and GOT2 expression and the relapse and OS of patients with CRC (lower panel). **f** Kaplan–Meier analysis of the associations between ASNS expression and the relapse and OS of patients with CRC (upper panel). Kaplan–Meier analysis of the associations between simultaneous SOX12 and ASNS expression and the relapse and OS of patients with CRC (lower panel)

Clinical associations between SOX12 and GLS, GOT2, ASNS, or HIF-1α expression in CRC tissues were further assessed. IHC staining and correlation analyses revealed positive correlations between SOX12 expression and HIF-1α, GLS, GOT2, and ASNS levels (Fig. 8a, b). Overexpression of HIF-1α, GLS, GOT2, and ASNS correlated with poor prognosis (Fig. 8c–f and Supplementary Table S2) and malignant characteristics (Supplementary Tables S5 and S6). Patients coexpressing SOX12 and HIF-1α, SOX12 and GLS, SOX12 and GOT2, or SOX12 and ASNS showed the highest recurrence rates and the lowest OS rates according to Kaplan–Meier analysis (Fig. 8c–f), suggesting that the HIF-1α/SOX12/GLS/GOT2/ASNS pathway contributes to CRC progression and can predict poor prognosis.

## Discussion

SOX12 has been characterized as an oncogene in various cancers to promote multiple malignant processes, including cell proliferation, survival, migration, and invasion^16,17,38,39^. As shown in the previous study, SOX12, a direct target of FOXQ1, promotes hepatocellular carcinoma (HCC) metastasis by upregulating Twist1 and FGFBP1^16^. SOX12 upregulation has also been reported to be associated with HCC metastasis and to increase cell cycle-dependent kinase 4 and insulin-like growth factor 2 mRNA-binding protein 1 expression^31^. In contrast, SOX12 silencing suppresses the proliferation and metastasis of breast cancer and lung cancer^40,41^. In this study, we found SOX12 overexpression to be associated with aggressive CRC cell characteristics and poor patient outcomes. SOX12 expression was an independent and significant risk factor for disease recurrence and reduced patient survival after curative resection. SOX12 was expressed at much higher levels in patients with metastasis and recurrence than in patients without metastasis or recurrence, and SOX12 overexpression indicated a poor prognosis. Ectopic expression of SOX12 promoted CRC cell proliferation and metastasis. These data strongly suggest that SOX12 contributes to malignant progression in CRC. Interestingly, Duquet et al.^18^ reported increased metastasis of HT29 and CC14 cells upon SOX12 knockdown. To address the contradictory results on the role of SOX12 in CRC, we silenced SOX12 expression in 17 lines of CRC cells and found that SOX12 knockdown suppressed the migration of 14/17 of CRC cell lines (Supplementary Figure S4). However, only 3/17 CRC cell lines displayed increased migration after SOX12 knockdown, including the CC14 and HT29 cell lines (Supplementary Figure S4), which is consistent with the results reported by Duquet et al.^18^. This discrepancy may be due to the different genetic backgrounds of the cells, such as gene mutations and virus infections; this possibility requires further exploration.

Aspartate was recently reported to be an endogenous metabolite limiting tumor growth^24^. Asparagine bioavailability drives breast cancer metastasis and protects tumor cells from glutamine depletion-induced cell death^25,42^. With regard to CRC, a study screening biomarkers of metabolic reprogramming in mouse and human CRC reported significantly increased levels of amino acid metabolites, including glutamic acid, proline, and arginine^23^, implying that amino acid disturbances might have a key role in CRC progression. However, the mechanism underlying the alterations in amino acid metabolism in CRC remains unclear. In this study, we used an Amino Acid Metabolism RT^2^ Profiler PCR Array with SOX12-overexpressing CRC cells and found that SOX12 transactivated GLS, GOT2, and ASNS expression to promote asparagine synthesis and CRC proliferation and metastasis. Asparagine, the side chain of which forms hydrogen bonds with the peptide backbone, is essential for protein synthesis. In the process of asparagine synthesis, oxaloacetate is converted to aspartate by GOT2, which transfers the amino group from glutamate to oxaloacetate, producing aspartate and α-ketoglutarate. Then, ASNS produces asparagine, glutamate, AMP, and pyrophosphate from aspartate, ATP, and glutamine. GLS, an amidohydrolase enzyme that generates glutamate from glutamine, is also required for intercellular asparagine production. Based on accumulating evidence, ASNS, GOT2, and GLS, as well as asparagine, substantially contribute to tumor growth and metastasis^33,43–48^. In our study, GLS, GOT2, and ASNS were found to be direct transcriptional targets of SOX12. SOX12 promoted asparagine synthesis by upregulating expression of GLS, GOT2, and ASNS, thus increasing CRC progression. Silencing of GLS, GOT2, and ASNS dramatically reduced SOX12-mediated CRC cell proliferation, migration, and invasion, whereas GLS, GOT2, and ASNS overexpression attenuated the decrease in CRC cell aggressiveness induced by SOX12 knockdown. Moreover, treatment of cells with l-asparaginase, which degrades asparagine and is used to treat leukemia, significantly reduced SOX12-induced CRC cell proliferation and metastasis, representing a potential therapeutic treatment for patients with CRC. Furthermore, GLS, GOT2, and ASNS expression was upregulated in human CRC tissues compared with adjacent nontumor tissues and this overexpression was correlated with more aggressive clinical features. SOX12 expression was positively correlated with GLS, GOT2, and ASNS levels, and coexpression was correlated with a worse prognosis. Collectively, these findings indicate a novel mechanism by which the SOX12/GLS/GOT2/ASNS axis regulates metabolism to generate asparagine and fuel CRC progression.

Given our finding that SOX12-mediated asparagine synthesis is required for CRC proliferation and metastasis, we further sought to determine the mechanism by which SOX12 and amino acid metabolism are dysregulated in CRC. Extensive studies have shown that the tumor microenvironment, particularly hypoxia, is a key driver of metabolic reprogramming that impact cell survival and metastasis^35,36^. Recently, increasing lines of evidence have revealed that the hypoxia response, particularly its master regulator HIF-1, regulates amino acid metabolism in cancer cells^49^. Notably, Nijhuis et al.^37^ reported that hypoxia determines the regulation and utilization of amino acid metabolism in CRC cells, and that alanine, aspartate, and glutamate metabolism is significantly increased in hypoxic cells. However, whether hypoxia contributes to SOX12-mediated asparagine dysregulation in human CRC remains unclear. In this study, we identified five potential HIF-1α-binding sites in the SOX12 promoter. Subsequent luciferase reporter assays with serial deletion constructs produced by site-directed mutagenesis, as well as ChIP, western blotting, and PCR analyses, indicated that HIF-1α transactivated SOX12 by directly binding to the fourth HIF-1α-binding site within the SOX12 promoter. In addition, based on the results of clinical investigations, HIF-1α expression was associated with SOX12 expression, and coexpression of these two molecules predicted a poor prognosis for patients with CRC. Together, these findings indicate that the hypoxia-related protein HIF-1α functions as a transcriptional regulator of SOX12, which promotes asparagine synthesis, proliferation, and metastasis in CRC cells, providing evidence for the promotion of human cancer progression by dysregulated amino acid metabolism under hypoxic conditions.

In summary, SOX12, a direct target of HIF-1α, is significantly upregulated in CRC and correlates with a poor prognosis. SOX12 overexpression promotes CRC cell proliferation and metastasis through transactivation of GLS, GOT2, and ASNS expression, thus contributing to asparagine synthesis during CRC development. l-Asparaginase treatment significantly suppresses SOX12-mediated CRC cell proliferation and metastasis. In conclusion, this study reports a new function of SOX12 in CRC progression, implicating SOX12 as a potentially useful prognostic biomarker for the development of an effective treatment for CRC.

## Materials and methods

### ChIP assay

ChIP assays were performed with a Magna ChIP G assay kit (EMD Millipore, Temecula, CA, USA). Briefly, cells were crosslinked with 1% formaldehyde for 10 min at 37 °C and then quenched with glycine. The bound DNA was coimmunoprecipitated from the sonicated cell lysates (6 rounds of 15 s on and 90 s off) with primary antibodies against SOX12, HIF-1α, and normal IgG (negative control, NC) (Cell Signaling Technology, Danvers, MA, USA) and subjected to PCR to amplify the corresponding binding sites on the promoters (see Supplementary Table S7 for the primer sequences). The experiments were independently repeated at least three times.

### Luciferase reporter assay

Luciferase activity in various treated cells was measured using a Dual Luciferase Assay Kit (Promega, USA) according to the manufacturer’s instructions. We added lysis buffer to culture dishes, to lyse the transfected cells, and centrifuged the resulting lysate for 1 min in an Eppendorf microcentrifuge at maximum speed. Relative luciferase activity was measured using a Modulus^TM^ TD20/20 Luminometer (Turner Biosystems, USA) and the transfection efficiencies were normalized based on *Renilla* luciferase activity. The experiments were independently repeated at least three times.

### Cell culture

Human CRC cells (SW480, SW1116, DLD-1, HT-29, RKO, Caco-2, SW48, HCT-15, HCT116, SW620, Colo320, LoVo, Colo201, Colo205, T84, and SK-CO-1 cells) were purchased from American Type Culture Collection. The cells were cultured in Dulbecco’s modified Eagle’s medium (Gibco, Thermo Fisher Scientific, Cambridge, MA, USA) supplemented with 10% fetal bovine serum (FBS, Gibco), 100 μg/ml penicillin, and 100 μg/ml streptomycin (Gibco) in a 5% CO_2_ atmosphere at 37 °C.

### Plasmid construction

Plasmid vectors were constructed using standard procedures and the PCR primers are shown in Supplementary Table S7. The *SOX12* promoter sequence (− 1526/ + 28) was obtained from human genomic DNA using PCR. This sequence is located at the position of the transcriptional start site (− 1526 to + 28) in the 5′-flanking region of the human *SOX12* gene. The vector was constructed by incorporating forward and reverse primers at the 5′- and 3′-ends of the KpnI and HindIII sites, respectively. The PCR products were inserted between the digested KpnI and HindIII sites of the pGL3-Basic vector (Promega). Similarly, 5′-flanking region deletion mutants of the *SOX12* promoter ((− 1526/ + 28) SOX12, (− 1357/ + 28) SOX12, (− 809/ + 28) SOX12, (− 708/ + 28) SOX12, and (−428/ + 28) SOX12) were produced using the (− 1526/ + 28) SOX12 vector as a template. A QuikChange II Site-Directed Mutagenesis Kit (Stratagene, La Jolla, CA, USA) was used to mutate the HIF-1α-binding sites in the SOX12 promoter. DNA sequencing was used to verify the successful construction of the vectors. Other promoter vectors were constructed using similar methods.

### In vivo metastasis model and bioluminescence imaging (BLI)

All animal procedures were approved by the Committee on the Use of Live Animals in Teaching and Research of the Fourth Military Medical University. BALB/C nude mice (6–8 weeks old) were treated based on the institutional guidelines for animal care and were housed in a standard specific pathogen-free environment. A metastatic CRC mouse model was established according to an existing protocol. Briefly, 4 × 10^6^ cells resuspended in 100 μl of phosphate-buffered saline (PBS) were injected into the tail veins (for lung metastasis) or the spleens (for liver metastasis) of nude mice. For the in vivo tracking assay, firefly luciferase was transfected into different groups of cells and tumor formation and metastasis were examined using in vivo BLI. The mice were intraperitoneally (i.p.) injected with 100 mg/kg d-luciferin (Xenogen, Hopkinton, MA, USA) and bioluminescence signals were detected using an IVIS 100 Imaging System (Xenogen). We first acquired a photograph of each mouse and then performed BLI after various durations (1–60 s). IVIS Living Image (Xenogen) software was used to superimpose the resulting grayscale and pseudocolor images, and match the observed luciferase signals to their locations in the mice. The survival of all mice was recorded throughout the experiment. Mice were killed after 9 weeks, and the lungs and liver were removed for standard histological examinations.

### In vivo tumor growth in the xenograft model

All animal experiments were approved by the Institutional Animal Care and Use Committee of the Fourth Military Medical University. An in vivo tumorigenesis xenograft model was established in 6- to 8-week-old BALB/c athymic nude mice. Suspended treated cells were subcutaneously injected into the flank of each mouse (10 mice per group, 6 × 10^6^ cells in 150 μl of PBS per mouse). The mice were weighed and the tumor size was measured using vernier calipers. The tumor volume was calculated using the following equation: maximum tumor diameter (*L*) × diameter at a right angle to that axis (*W*)^2^/2. The mice were randomly divided into control and treatment groups when the tumors reached ~100 mm^3^. l-Asparaginase was administered at a dose of 5 UI/g daily via i.p. injection. Subsequently, the tumor-bearing mice underwent in vivo BLI (IVIS, Xenogen). After 4 weeks, the mice were killed according to the institutional ethical guidelines; then, the mice were weighed and the tumor size was measured. The tumors were then embedded in paraffin and prepared for H&E staining. The sample size was determined based on experimental pilot studies with a power of 0.8 and a type 1 error (*α*) of 0.05. After the mice were killed, the relevant tests and data analyses were performed by two independent investigators who were blinded to the groups.

### Patients and follow-up

The patient study was approved by the Ethics Committee of the Fourth Military Medical University. All patients provided written informed consent for participation in the study. For cohort I, we recruited 390 adult patients with CRC, who underwent surgical resection between January 2005 and December 2007 at the Tongji Hospital of Tongji Medical College (Wuhan, China). From January 2005 to December 2007, we obtained fresh CRC specimens and adjacent tissues from 363 adult patients (cohort II) who underwent surgery at Xijing Hospital, Fourth Military Medical University (Xi’an, China). No patients enrolled in the cohorts received any preoperative chemotherapy or radiotherapy. Tumor pathological staging was based on AJCC and International Union Against Cancer criteria. Patients with stage II, III, and IV tumors received adjuvant chemotherapy after surgery and no patients received postoperative radiotherapy. H&E staining performed by the Department of Pathology, Xijing Hospital, confirmed the histomorphology of all primary tumor specimens and regional lymph nodes. Twenty normal colonic epithelial tissues and 140 pairs of fresh-frozen CRC tissues and peripheral nontumor tissues were collected and stored in liquid nitrogen after surgical resection. RNA was extracted from these tissues to assess the expression of SOX12 mRNA. Six normal colonic epithelial tissues and 20 fresh-frozen CRC tissues were collected after surgical resection for use in ChIP assays.

Imaging methods were used to diagnose recurrence and distant metastases during at least 8 years of complete follow-up, including computed tomography, endoscopy, positron emission tomography, ultrasonography, magnetic resonance imaging, and, in some cases, cytological analyses and biopsy. The time from surgery to the first occurrence of any of the following events was defined as the disease-free survival time: CRC recurrence; CRC distant metastasis; second noncolorectal malignancy, excluding basal cell carcinoma of the skin and carcinoma in situ of the cervix; or any cause of death without a record of cancer-related events. The time elapsed from surgery to the death of patients with CRC was defined as the OS time. Telephone inquiries and questionnaires were used to update the follow-up information of all participants every 3 months. Patient deaths were confirmed by family reports and review of public records.

### Construction of tissue microarrays and IHC staining

We used a tissue microarray (Shanghai Biochip, Shanghai, China) to create chips of CRC samples and corresponding adjacent colorectal tissues. The tissue microarray was stained with antibodies against SOX12 (Sigma-Aldrich Corporation, Los Angeles, CA, USA, SAB4502835), HIF-1α (Abcam, Cambridge, MA, USA, ab1), GLS (Abcam, ab156876), GOT2 (Abcam, ab153924), and ASNS (Abcam, ab126254). The staining intensity of the entire section and the protein expression levels in the array were independently scored by two pathologists. Based on the manufacturer’s instructions, IHC staining was performed using the Dako Envision Plus System (Dako, Carpinteria, CA, USA).

Two independent observers, who were blinded to the clinical outcomes, analyzed the data. The staining intensity was scored as 0 (negative), 1 (weak), or 2 (strong). The degree of staining was scored based on the percentage of positive cells as follows: 0 (0%), 1 (1–25%), 2 (26–50%), 3 (51–75%), and 4 (76–100%). The staining intensity and degree scores were multiplied to determine the final score (negative or positive) for each sample. A final score of ≤ 3 points for a sample (0, 1, 2, 3) was considered negative and a final score of ≥ 4 points (4, 6, 8) was considered positive.

### Construction of the lentivirus and stable cell lines

Lentiviral vectors containing short hairpin RNA (shRNA) sequences were constructed using the pLKO.1-TRC (Addgene, Cambridge, MA, USA) vector and designated LV-shSOX12, LV-shGLS, LV-shGOT2, LV-shASNS, and LV-shcontrol. LV-shcontrol was a nontargeting shRNA control. The pLKO.1-puro Non-Target shRNA Control Plasmid DNA vector (Sigma, SHC016) contains an shRNA insert that does not target any known genes from any species. The shRNA sequences are listed in Supplementary Table S8. Lentiviral vectors carrying the human SOX12, GLS, GOT2, and ASNS genes were constructed in FUW-tetO (Addgene) and designated LV-SOX12, LV-GLS, LV-GOT2, and LV-ASNS, respectively. We used an empty vector as a NC and defined it as LV-control.

Concentrated lentiviruses were transfected into CRC cells in the presence of polybrene (6 μg/ml) with a multiplicity of infection ranging from 30 to 50. At 72 h post infection, CRC cells were incubated with 2.5 μg/ml puromycin (OriGene, MD, USA) for 2 weeks. Screened cells with silenced and upregulated expression were used in subsequent assays.

### Transient transfection

Cells were cultured in a 24-well plate at a density of 1 × 10^5^ cells/well. After 12–24 h, the cells were transfected with 0.02 μg of the pRL-TK plasmid, 0.18 μg of the promoter reporter plasmid, and 0.6 μg of the expression vector plasmid using Lipofectamine 2000 (Invitrogen, Thermo Fisher Scientific, Cambridge, MA, USA) according to the manufacturer’s instructions. Five hours later, the cells were washed with fresh medium and allowed to recover for 48 h in fresh medium containing 1% FBS. The cells were starved in serum-free medium before use in the assays.

### Quantitative reverse transcription PCR

According to the manufacturer’s instructions, total RNA was extracted from cells or tissues using TRIzol reagent (Invitrogen), followed by reverse transcription performed using the Advantage RT-for-PCR Kit (Takara, Dalian, China). Aliquots of double-stranded cDNA were amplified using a SYBR Green PCR Kit (Applied Biosystems, Thermo Fisher Scientific, Cambridge, MA, USA) for qRT-PCR and melting curve analysis was performed. The reaction conditions were as follows (45 cycles): 95 °C for 15 s, 55–60 °C for 15 s, and 72 °C for 15 s. The Ct value was detected during the exponential amplification phase of qRT-PCR and the amplification curves were analyzed using SDS 1.9.1 software (Applied Biosystems). The relative expression levels of the target genes (defined as the fold change) in the cell lines were calculated using the 2^–ΔΔCt^ method with the following equations: ΔCt = ΔCt^target^ – ΔCt^GAPDH^ and ΔΔCt = ΔCt^expressing vector^ – ΔCt^control vector^. We normalized the expression to the fold change detected in matching control cells, which was set to 1.0. We determined the fold changes in the expression of the target genes in clinical tissue samples using the 2^–ΔΔCt^ method with the following equation: ΔΔCt = ΔCt^tumor^ – ΔCt^nontumor^. We normalized the value to the mean fold change in the normal colorectal tissues, which was set to 1.0. All reactions were repeated and the primer sequences are listed in Supplementary Table S7.

### Western blotting analysis

Proteins extracted from cells were separated by SDS-polyacrylamide gel electrophoresis and then transferred to nitrocellulose membranes. Nonspecific binding to the membranes was blocked with 5% skim milk in TBST (150 mM NaCl, 120 mM Tris-HCl pH 7.4, and 0.05% Tween 20) at room temperature for 1 h and the membranes were then incubated with specific antibodies overnight at 4 °C. β-Actin expression was used as an internal control. The anti-β-actin antibody (sc-47778) was purchased from Santa Cruz Biotechnology (Santa Cruz, CA, USA). Antibodies against SOX12 (SAB 1411973), GLS (ab156876), GOT2 (ab153924), and ASNS (ab126254) were purchased from Abcam (Cambridge, MA, USA). Antibodies against HIF-1α (36169) were purchased from Cell Signaling Technology (Beverly, MA, USA). The membranes were carefully washed with TBST three times and then incubated with horseradish peroxidase-conjugated secondary antibodies diluted in TBST. The protein expression levels were detected by chemiluminescence using Dura SuperSignal Substrate (Pierce, Thermo Fisher Scientific, Cambridge, MA, USA). The experiments were repeated independently at least three times.

### Agents

l-Asparaginase (ENZ-287) was purchased from ProSpec (Rehovot, Israel). All agents were used according to the manufacturers’ instructions.

### CCK-8 assay

For the CCK-8 assay, cells were seeded into 96-well plates at a density of 1000 cells in 100 μl of complete medium per well. At each time point, the original medium was replaced with CCK-8 solution (TransDetect Cell Counting Kit, Transgene, Beijing, China) and complete medium mixed at a 1:9 ratio, and the cells were then incubated at 37 °C for 2 h. The absorbance of each sample was recorded at 450 nm using a microplate reader (Tecan Group, Ltd, Zürich, Switzerland) and each sample was measured three times.

### Colony formation assay

Transfected cells (1000 per well) were cultured in 6-well plates. After 14–18 days of culture, the cells formed stable colonies. The cell colonies were fixed with 70% ethanol and then stained with a crystal violet solution. Colonies containing more than 50 cells were counted and each group included three replicates.

### Wound-healing assay

Cells were cultured in a 12-well plate until they reached 90% confluence and a wound was then created with a sterile tip. The suspended cells were removed by washing the plate with PBS and the remaining adherent cells were cultured in minimum essential medium supplemented with 5% FBS. The cells were imaged at 0, 24, and 48 h, and the wound-healing rate (%) was evaluated using TScratch software (Computational Science & Engineering Laboratory, Zürich, Switzerland). The experiments were repeated independently at least three times.

### In vitro migration and invasion assays

The migration and invasion abilities of each cell line were measured using 24-well Transwells (8 μm pore size, Corning, Inc., NY, USA). In the Transwell migration experiment, 5 × 10^4^ cells were seeded in the top chamber lined with an uncoated membrane. In the invasion experiment, chamber inserts were coated with 200 mg/ml Matrigel and dried overnight under sterile conditions. Then, 1 × 10^5^ cells were plated in the upper chamber and the numbers of cells invading and migrating to the lower layer were counted 48 h later. Each experimental group included three replicates.

### Metabolic analysis

Cells were washed twice with PBS and the metabolites in the cells were extracted on dry ice with a mixture of acetonitrile, water, and formic acid (80:19:1, v/v/v). The cells were then detached, subjected to two freeze–thaw cycles, and centrifuged. The residue was re-extracted with methanol and the supernatants were combined and evaporated under a vacuum. The levels of U-^13^C_5_-labeled metabolites in the indicated cells were determined using LC-MS/MS according to the manufacturer’s instructions. The experiments were repeated independently at least three times.

### Datasets

TCGA (https://cancergenome.nih.gov) datasets were used to determine the expression of SOX12 mRNA in human cancer specimens compared to normal tissues.

### Statistical analysis

All analyses were performed using SPSS (version 18.0) software. Quantitative data were compared between groups using Student’s *t*-test. Classification data were analyzed using Fisher’s exact test. The Cox proportional hazard model was used to identify independent factors affecting survival and recurrence based on variables selected from univariate analysis. Cumulative recurrence and survival rates were determined using the Kaplan–Meier method and a log-rank test. *P* < 0.05 was considered to represent a significant difference.

## Supplementary information

Supplementary Figure S1

Supplementary Figure S2

Supplementary Figure S3

Supplementary Figure S4

Supplementary Table S1

Supplementary Table S2

Supplementary Table S3

Supplementary Table S4

Supplementary Table S5

Supplementary Table S6

Supplementary Table S7

Supplementary Table S8

Supplemental Figure legends
