# Supplementary figures and images for: SOX12 promotes colorectal cancer cell proliferation and metastasis by regulating asparagine synthesis
Source: Cell Death Dis. 2019 Mar 11;10(3):239. doi: 10.1038/s41419-019-1481-9 (PMC6412063; doi:10.1038/s41419-019-1481-9)

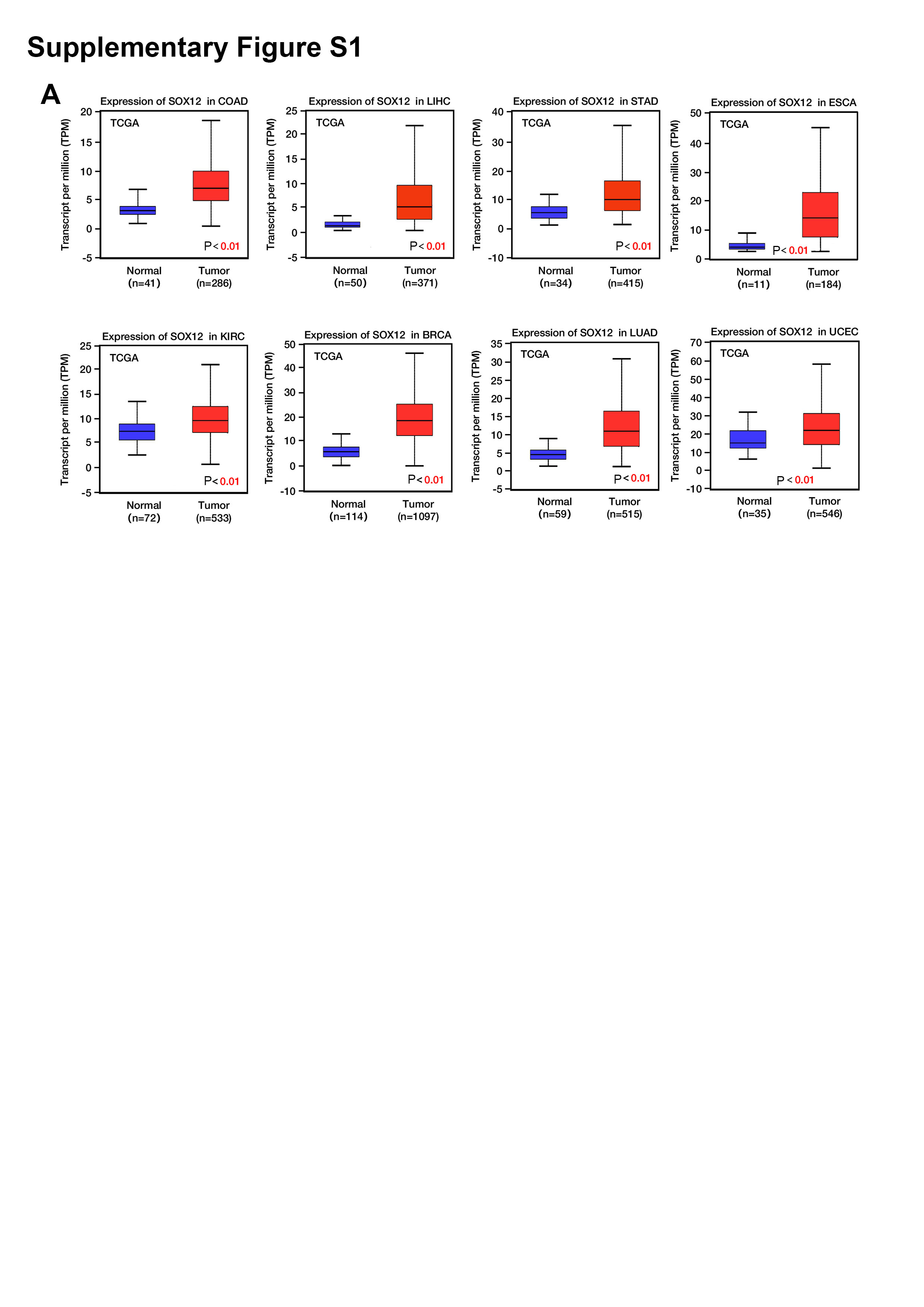

Supplement: Supplementary file 1 — Supplementary Figure S1 [file 41419_2019_1481_MOESM1_ESM.jpg]

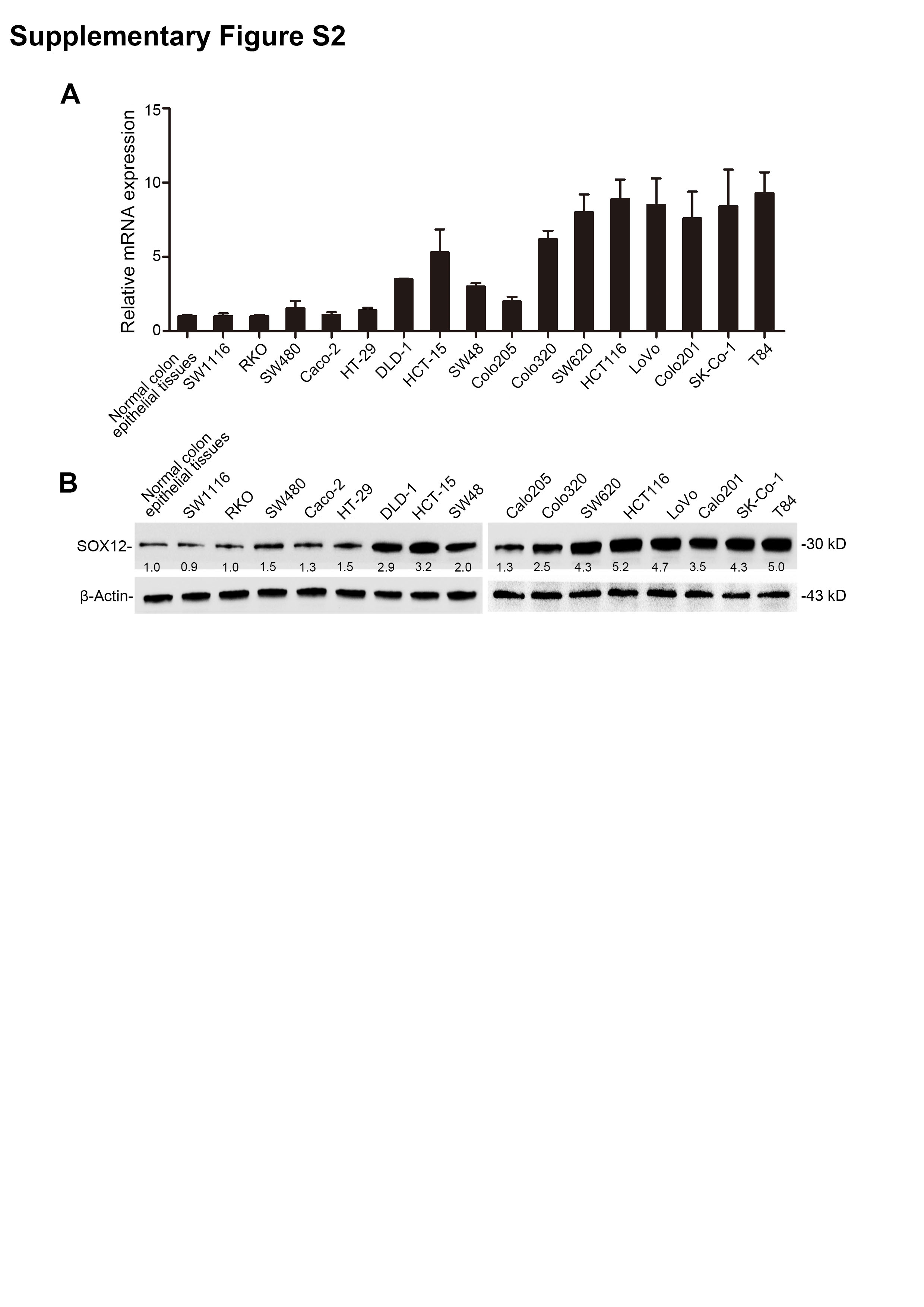

Supplement: Supplementary file 2 — Supplementary Figure S2 [file 41419_2019_1481_MOESM2_ESM.jpg]

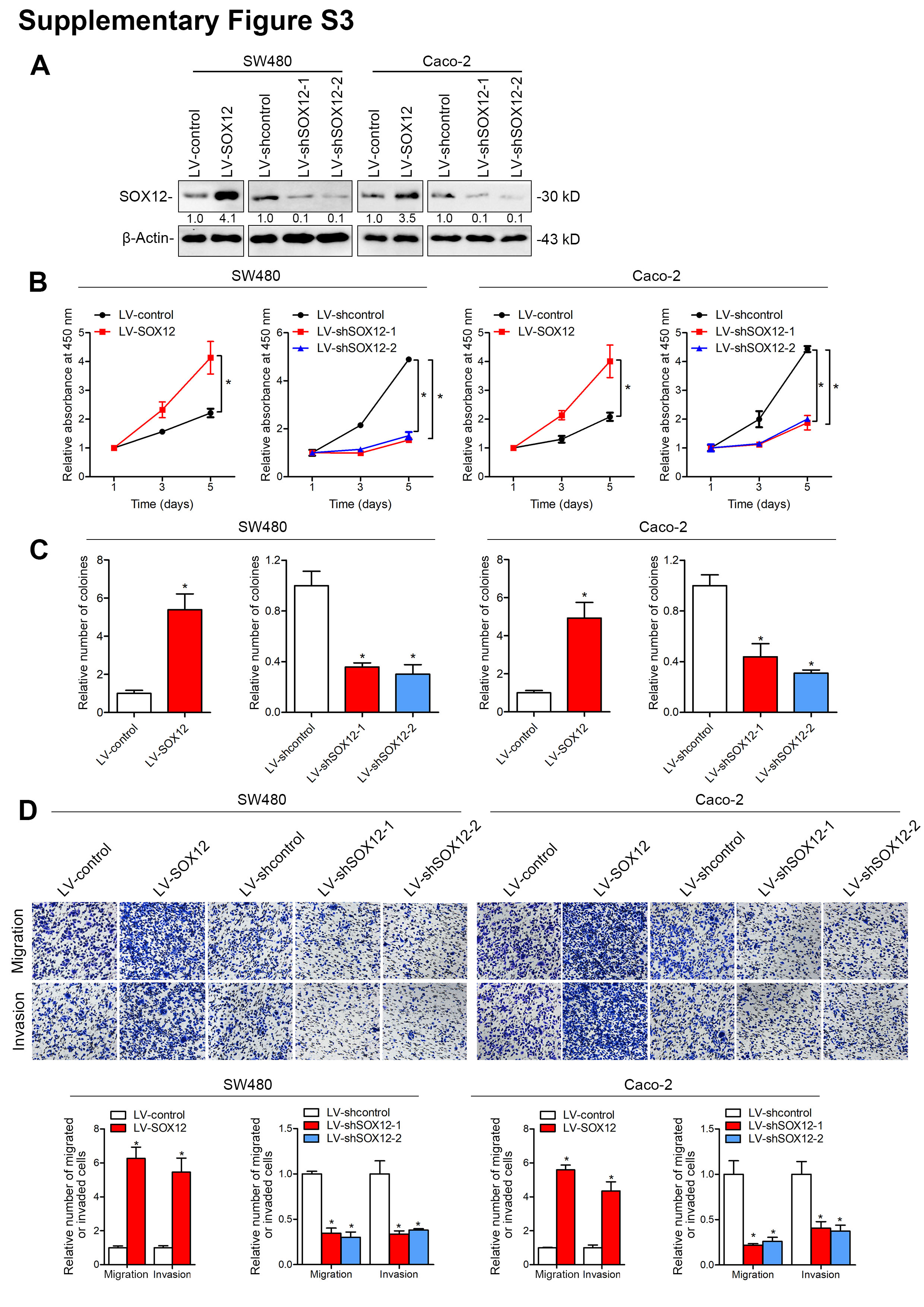

Supplement: Supplementary file 3 — Supplementary Figure S3 [file 41419_2019_1481_MOESM3_ESM.jpg]

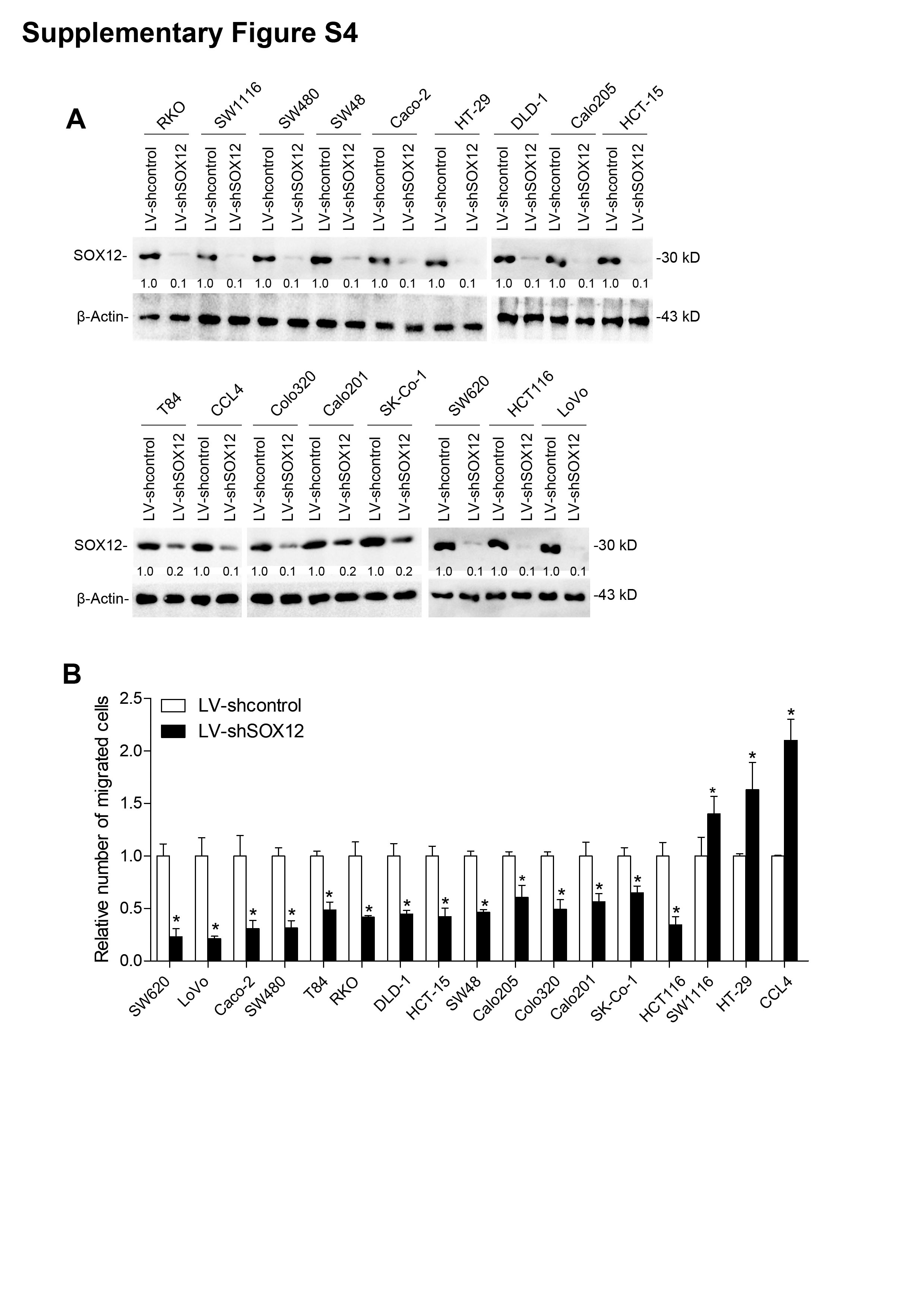

Supplement: Supplementary file 4 — Supplementary Figure S4 [file 41419_2019_1481_MOESM4_ESM.jpg]
