# Supplementary material for: SOX12 promotes colorectal cancer cell proliferation and metastasis by regulating asparagine synthesis
Source: Cell Death Dis. 2019 Mar 11;10(3):239. doi: 10.1038/s41419-019-1481-9 (PMC6412063; doi:10.1038/s41419-019-1481-9)
Supplement: Supplementary file 5 — Supplementary Table S1 [file 41419_2019_1481_MOESM5_ESM.docx]

Supplementary Table S1. Correlation between SOX12 expression and clinicopathological characteristics in two independent cohorts of human CRC tissues

|  |  | Cohort I (n=390) | | P value |  | Cohort II (n=363) | | P value |
| --- | --- | --- | --- | --- | --- | --- | --- | --- |
| Clinicopathological variables | | Tumor SOX12 expression | |  |  | Tumor SOX12 expression | |  |
|  |  | Negative (n=216) | Positive (n=174) |  |  | Negative (n=228) | Positive (n=135) |  |
| Age | | 68.48(10.39) | 66.26(12.78) | 0.059 |  | 66.43(11.41) | 66.07(11.25) | 0.889 |
| Sex | female | 93 | 84 | 0.308 |  | 91 | 70 | 0.029 |
|  | male | 123 | 90 |  |  | 137 | 65 |  |
| Tumor location | right colon | 86 | 79 | 0.257 |  | 112 | 61 | 0.328 |
|  | left colon | 96 | 72 |  |  | 67 | 61 |  |
|  | rectum | 34 | 23 |  |  | 49 | 13 |  |
| Tumor size | ＜5cm | 86 | 63 | 0.529 |  | 101 | 53 | 0.38 |
|  | ≥5cm | 130 | 111 |  |  | 127 | 82 |  |
| Tumor differentiation | well or moderate | 161 | 63 | <0.001 |  | 186 | 64 | <0.001 |
|  | poor | 55 | 111 |  |  | 42 | 71 |  |
| Tumor invasion | T1 | 14 | 2 | 0.009 |  | 4 | 3 | <0.001 |
|  | T2 | 15 | 9 |  |  | 30 | 0 |  |
|  | T3 | 149 | 124 |  |  | 153 | 89 |  |
|  | T4 | 38 | 39 |  |  | 41 | 43 |  |
| Lymph node metastasis | absent | 190 | 36 | <0.001 |  | 159 | 40 | <0.001 |
|  | present | 26 | 138 |  |  | 69 | 95 |  |
| Distant metastasis | absent | 211 | 107 | <0.001 |  | 209 | 89 | <0.001 |
|  | present | 5 | 67 |  |  | 19 | 46 |  |
| AJCC stage | Stage I | 18 | 2 | <0.001 |  | 33 | 2 | <0.001 |
|  | Stage II | 171 | 28 |  |  | 123 | 36 |  |
|  | Stage III | 22 | 79 |  |  | 53 | 51 |  |
|  | Stage IV | 5 | 65 |  |  | 19 | 46 |  |
