# Supplementary material for: SOX12 promotes colorectal cancer cell proliferation and metastasis by regulating asparagine synthesis
Source: Cell Death Dis. 2019 Mar 11;10(3):239. doi: 10.1038/s41419-019-1481-9 (PMC6412063; doi:10.1038/s41419-019-1481-9)
Supplement: Supplementary file 6 — Supplementary Table S2 [file 41419_2019_1481_MOESM6_ESM.doc]

Supplementary Table S2. Univariate and multivariate analysis of factors associated with survival and recurrence in two independent cohorts of human CRC tissues Cohort I (n=390)

|  | Recurrence | | | | | | |  | Survival | | | | | | |
| --- | --- | --- | --- | --- | --- | --- | --- | --- | --- | --- | --- | --- | --- | --- | --- |
| Variables | Univariate analysis | | |  | multivariate analysis | | |  | Univariate analysis | | |  | multivariate analysis | | |
|  | HR | 95% CI | p value |  | HR | 95% CI | P value |  | HR | 95% CI | p value |  | HR | 95% CI | P value |
| Age | 0.998 | 0.988-1.009 | 0.774 |  |  |  |  |  | 1.000 | 0.989-1.011 | 0.951 |  |  |  |  |
| Sex (female versus male) | 1.070 | 0.847-1.351 | 0.569 |  |  |  |  |  | 1.116 | 0.880-1.415 | 0.367 |  |  |  |  |
| Tumor size (≤5 versus >5 cm) | 0.901 | 0.709-1.146 | 0.396 |  |  |  |  |  | 0.877 | 0.686-1.122 | 0.297 |  |  |  |  |
| Tumor differentiation(well/moderate versus poor) | 0.469 | 0.370-0.593 | <0.001 |  | 0.818 | 0.627-1.067 | 0.139 |  | 0.449 | 0.353-0.571 | <0.001 |  | 0.824 | 0.628-1.083 | 0.165 |
| Tumor invasion(T1-T3 versus T4) | 0.605 | 0.461-0.796 | <0.001 |  | 0.795 | 0.598-1.059 | 0.117 |  | 0.607 | 0.459-0.803 | <0.001 |  | 0.801 | 0.598-1.072 | 0.136 |
| Lymph node metastasis (absent versus present) | 0.193 | 0.150-0.248 | <0.001 |  | 1.629 | 0.745-3.562 | 0.222 |  | 0.172 | 0.132-0.222 | <0.001 |  | 1.351 | 0.619-2.951 | 0.450 |
| Distant metastasis (absent versus present) | 0.130 | 0.095-0.178 | <0.001 |  | 0.486 | 0.342-0.689 | <0.001 |  | 0.111 | 0.081-0.154 | <0.001 |  | 0.419 | 0.293-0.598 | <0.001 |
| AJCC stage(I-II versus III-Ⅳ) | 0.179 | 0.138-0.230 | <0.001 |  | 0.253 | 0.111-0.575 | 0.001 |  | 0.159 | 0.122-0.207 | <0.001 |  | 0.272 | 0.120-0.619 | 0.002 |
| SOX12 expression (negative versus positive) | 0.261 | 0.204-0.333 | <0.001 |  | 0.635 | 0.452-0.890 | 0.008 |  | 0.237 | 0.185-0.305 | <0.001 |  | 0.593 | 0.421-0.834 | 0.003 |
| HIF-1α expression (negative versus positive) | 0.268 | 0.210-0.343 | <0.001 |  | 0.591 | 0.433-0.807 | 0.001 |  | 0.281 | 0.219-0.360 | <0.001 |  | 0.681 | 0.497-0.935 | 0.018 |
| GLS expression (negative versus positive) | 0.444 | 0.350-0.562 | <0.001 |  | 0.977 | 0.735-1.299 | 0.873 |  | 0.422 | 0.332-0.537 | <0.001 |  | 0.945 | 0.707-1.263 | 0.703 |
| GOT2 expression (negative versus positive) | 0.328 | 0.259-0.417 | <0.001 |  | 0.712 | 0.534-0.949 | 0.021 |  | 0.318 | 0.249-0.405 | <0.001 |  | 0.702 | 0.523-0.942 | 0.018 |
| ASNS expression (negative versus positive) | 0.318 | 0.250-0.404 | <0.001 |  | 0.573 | 0.433-0.758 | <0.001 |  | 0.307 | 0.240-0.392 | <0.001 |  | 0.588 | 0.441-0.784 | <0.001 |
