# Supplementary material for: SOX12 promotes colorectal cancer cell proliferation and metastasis by regulating asparagine synthesis
Source: Cell Death Dis. 2019 Mar 11;10(3):239. doi: 10.1038/s41419-019-1481-9 (PMC6412063; doi:10.1038/s41419-019-1481-9)
Supplement: Supplementary file 7 — Supplementary Table S3 [file 41419_2019_1481_MOESM7_ESM.doc]

Supplementary Table S3. Univariate and multivariate analysis of factors associated with survival and recurrence in two independent cohorts of human CRC tissues

Cohort II (n=363)

|  | Recurrence | | | | | | |  | Survival | | | | | | |
| --- | --- | --- | --- | --- | --- | --- | --- | --- | --- | --- | --- | --- | --- | --- | --- |
| Variables | Univariate analysis | | |  | multivariate analysis | | |  | Univariate analysis | | |  | multivariate analysis | | |
|  | HR | 95% CI | p value |  | HR | 95% CI | P value |  | HR | 95% CI | p value |  | HR | 95% CI | P value |
| Age | 0.999 | 0.986-1.011 | 0.819 |  |  |  |  |  | 0.998 | 0.985-1.011 | 0.772 |  |  |  |  |
| Sex (female versus male) | 1.272 | 0.964-1.677 | 0.089 |  |  |  |  |  | 1.212 | 0.916-1.604 | 0.179 |  |  |  |  |
| Tumor size (≤5 versus >5 cm) | 0.833 | 0.628-1.105 | 0.205 |  |  |  |  |  | 0.827 | 0.621-1.101 | 0.193 |  |  |  |  |
| Tumor differentiation(well/moderate versus poor) | 0.184 | 0.138-0.245 | <0.001 |  | 0.785 | 0.540-1.142 | 0.206 |  | 0.186 | 0.139-0.249 | <0.001 |  | 0.836 | 0.570-1.225 | 0.358 |
| Tumor invasion(T1-T3 versus T4) | 0.350 | 0.261-0.469 | <0.001 |  | 0.617 | 0.445-0.854 | 0.004 |  | 0.357 | 0.265-0.480 | <0.001 |  | 0.636 | 0.456-0.888 | 0.008 |
| Lymph node metastasis (absent versus present) | 0.138 | 0.100-0.190 | <0.001 |  | 0.465 | 0.159-1.356 | 0.161 |  | 0.136 | 0.098-0.189 | <0.001 |  | 0.421 | 0.142-1.250 | 0.119 |
| Distant metastasis (absent versus present) | 0.114 | 0.081-0.159 | <0.001 |  | 0.475 | 0.158-1.429 | 0.185 |  | 0.113 | 0.081-0.157 | <0.001 |  | 0.511 | 0.167-1.567 | 0.240 |
| AJCC stage(I-II versus III-Ⅳ) | 0.134 | 0.097-0.187 | <0.001 |  | 0.378 | 0.255-0.560 | <0.001 |  | 0.133 | 0.095-0.186 | <0.001 |  | 0.366 | 0.246-0.546 | <0.001 |
| SOX12 expression (negative versus positive) | 0.306 | 0.231-0.406 | <0.001 |  | 0.567 | 0.420-0.767 | <0.001* |  | 0.304 | 0.228-0.404 | <0.001 |  | 0.584 | 0.429-0.793 | 0.001* |
