# Supplementary material for: SOX12 promotes colorectal cancer cell proliferation and metastasis by regulating asparagine synthesis
Source: Cell Death Dis. 2019 Mar 11;10(3):239. doi: 10.1038/s41419-019-1481-9 (PMC6412063; doi:10.1038/s41419-019-1481-9)
Supplement: Supplementary file 8 — Supplementary Table S4 [file 41419_2019_1481_MOESM8_ESM.docx]

Supplementary Table S4. List of genes differentially expressed in SW480-SOX12 versus SW480-control cells using a human amino acid metabolism PCR array

| **Symbol** | **Fold change** | **Description** |
| --- | --- | --- |
| ASNS | 6.055311823 | Asparagine synthetase (glutamine-hydrolyzing) |
| GLS | 5.921000095 | Glutaminase |
| GOT2 | 5.129721366 | Glutamic-oxaloacetic transaminase 2, mitochondrial (aspartate aminotransferase2) |
| IDO1 | 4.903557812 | Indoleamine 2,3-dioxygenase 1 |
| GCDH | 4.891720967 | Glutaryl-CoA dehydrogenase |
| AMD1 | 4.865161722 | Adenosylmethionine decarboxylase 1 |
| CBS | 4.832620714 | Cystathionine-beta-synthase |
| PSAT1 | 4.812092009 | Phosphoserine aminotransferase 1 |
| HMGCS1 | 4.697527465 | 3-hydroxy-3-methylglutaryl-CoA synthase 1 (soluble) |
| TH | 4.651455329 | Tyrosine hydroxylase |
| HDC | 4.576175641 | Histidine decarboxylase |
| COMT | 4.489166154 | Catechol-O-methyltransferase |
| PSPH | 4.451836741 | Phosphoserine phosphatase |
| MAOB | 4.405378888 | Monoamine oxidase B |
| DLD | 4.378732217 | Dihydrolipoamide dehydrogenase |
| CTH | 4.342184983 | Cystathionase (cystathionine gamma-lyase) |
| BCAT1 | 4.335349908 | Branched chain amino-acid transaminase 1, cytosolic |
| GNMT | 4.326722343 | Glycine N-methyltransferase |
| ODC1 | 4.290629284 | Ornithine decarboxylase 1 |
| AHCY | 4.270551031 | Adenosylhomocysteinase |
| TPH2 | 4.108490672 | Tryptophan hydroxylase 2 |
| PRODH | 4.065087645 | Proline dehydrogenase (oxidase) 1 |
| OAT | 4.018959802 | Ornithine aminotransferase |
| LAP3 | 4.007646354 | Leucine aminopeptidase 3 |
| APIP | 3.992746175 | APAF1 interacting protein |
| PHGDH | 3.96865168 | Phosphoglycerate dehydrogenase |
| CAD | 3.965007164 | Carbamoyl-phosphate synthetase 2, aspartate transcarbamylase, and dihydroorotase |
| AASDHPPT | 3.930964898 | Aminoadipate-semialdehyde dehydrogenase-phosphopantetheinyl transferase |
| GCDH | 3.911866535 | Glutaryl-CoA dehydrogenase |
| DNMT1 | 3.817546757 | DNMT1 DNA (cytosine-5-)-methyltransferase 1 |
| GAMT | 3.803244344 | Guanidinoacetate N-methyltransferase |
| ASH1L | 3.771732314 | Ash1 (absent, small, or homeotic)-like (Drosophila) |
| EHHADH | 3.759077757 | Enoyl-CoA, hydratase/3-hydroxyacyl CoA dehydrogenase |
| HIBCH | 3.741974394 | 3-hydroxyisobutyryl-CoA hydrolase |
| PPAT | 3.6635554 | Phosphoribosyl pyrophosphate amidotransferase |
| TYRP1 | 3.621487286 | Tyrosinase-related protein 1 |
| BCAT2 | 3.571051487 | Branched chain amino-acid transaminase 2, mitochondrial |
| SRR | 3.567922027 | Serine racemase |
| AUH | 3.52859521 | AU RNA binding protein/enoyl-CoA hydratase |
| ALDH4A1 | 3.406775195 | Aldehyde dehydrogenase 4 family, member A1 |
| ACAT1 | 3.29486415 | Acetyl-CoA acetyltransferase 1 |
| TAT | 3.21427803 | Tyrosine aminotransferase |
| PYCR1 | 2.940369793 | Pyrroline-5-carboxylate reductase 1 |
| PRDX6 | 2.896224315 | Peroxiredoxin 6 |
| MIF | 2.885780374 | Macrophage migration inhibitory factor (glycosylation-inhibiting factor) |
| KMO | 2.858394061 | Kynurenine 3-monooxygenase (kynurenine 3-hydroxylase) |
| LDHA | 2.854073444 | Lactate dehydrogenase A |
| ACADM | 2.824790683 | Acyl-CoA dehydrogenase, C-4 to C-12 straight chain |
| MCCC2 | 2.801700495 | Methylcrotonoyl-CoA carboxylase 2 (beta) |
| CPS1 | 2.6164638 | Carbamoyl-phosphate synthase 1, mitochondrial |
| OXCT2 | 2.477891317 | 3-oxoacid CoA transferase 2 |
| ALDH18A1 | 2.381514268 | Aldehyde dehydrogenase 18 family, member A1 |
| HIBADH | 2.354301454 | 3-hydroxyisobutyrate dehydrogenase |
| ADSL | 2.315426911 | Adenylosuccinate lyase |
| GFPT1 | 2.192145594 | Glutamine--fructose-6-phosphate transaminase 1 |
| MAOA | 2.17208389 | Monoamine oxidase A |
| GLUD1 | 2.171599174 | Glutamate dehydrogenase 1 |
| DLD | 2.059398356 | Dihydrolipoamide dehydrogenase |
| AGMAT | 2.032252348 | Adenosylhomocysteinase |
| PNMT | 2.00004676 | Phenylethanolamine N-methyltransferase |
| TYR | 1.891096542 | Tyrosinase (oculocutaneous albinism IA) |
| VARS2 | 1.814149814 | Valyl-tRNA synthetase 2, mitochondrial (putative) |
| PYCRL | 1.700509074 | Pyrroline-5-carboxylate reductase-like |
| GPT | 1.670930053 | Glutamic-pyruvate transaminase (alanine aminotransferase) |
| PLOD3 | 1.60493633 | Procollagen-lysine, 2-oxoglutarate 5-dioxygenase 3 |
| TDO2 | 1.541605143 | Tryptophan 2,3-dioxygenase |
| GLDC | 1.506333221 | Glycine dehydrogenase (decarboxylating) |
| MPST | 1.423840978 | Mercaptopyruvate sulfurtransferase |
| LARS | 1.385484352 | Leucyl-tRNA synthetase |
| ACADS | 1.369580176 | Acyl-CoA dehydrogenase, C-2 to C-3 short chain |
| WBSCR22 | 1.266300020 | Williams Beuren syndrome chromosome region 22 |
| P4HA1 | 1.257191376 | Prolyl 4-hydroxylase, alpha polypeptide I |
| SHMT2 | 1.232616060 | Serine hydroxymethyltransferase 2 (mitochondrial) |
| ASS1 | 1.199074140 | Argininosuccinate synthase 1 |
| NOS2 | 1.098625823 | Nitric oxide synthase 2, inducible/Inos |
| ARG2 | 1.094307802 | Arginase, type II |
| ACADSB | 1.073399707 | Acyl-CoA dehydrogenase, short/branched chain |
| FTCD | 1.072555727 | Formiminotransferase cyclodeaminase |
| BCKDHA | 1.070782778 | Branched chain keto acid dehydrogenase E1, alpha polypeptide |
| ADSS | 1.068766150 | Adenylosuccinate synthase |
| CKB | 1.058762161 | Creatine kinase, brain |
| OTC | 1.048693440 | Ornithine carbamoyltransferase |
| HGD | 1.038624259 | Homogentisate 1,2-dioxygenase |
| MCEE | 1.028596947 | Methylmalonyl CoA epimerase |
| ALDH9A1 | 1.028102419 | Aldehyde dehydrogenase 9 family, member A1 |
| GOT1 | 1.018373054 | Glutamic-oxaloacetic transaminase 1, soluble (aspartate aminotransferase 1) |
| PIPOX | 1.018279252 | Pipecolic acid oxidase |
| AOC3 | 1.016820304 | Amine oxidase, copper containing 3 (vascular adhesion protein 1) |
| ALDH3B1 | 1.014201701 | Aldehyde dehydrogenase 3 family, member B1 |
| DBH | 1.013800102 | Dopamine beta-hydroxylase (dopamine beta-monooxygenase) |
| ABP1/AOC1 | 1.012788944 | Amiloride binding protein 1 (amine oxidase (copper-containing) |
| AASS | 1.007870889 | Aminoadipate-semialdehyde synthase |
| ALDH5A1 | -1.077470182 | Aldehyde dehydrogenase 5 family, member A1 |
| CYP1B1 | -1.086140339 | Cytochrome P450, family 1, subfamily B, polypeptide 1 |
| CHDH | -1.096051487 | Choline dehydrogenase |
| CAT | -1.105526808 | Catalase |
| ACY1 | -1.155241699 | Aminoacylase 1 |
| TPO | -1.159200715 | Thyroid peroxidase |
| AMT | -1.169468083 | Aminomethyltransferase |
| MTR | -1.169937202 | 5-methyltetrahydrofolate-homocysteine methyltransferase |
| PCCA | -1.175217630 | Propionyl CoA carboxylase, alpha polypeptide |
| DDC | -1.179437682 | Dopa decarboxylase (aromatic L-amino acid decarboxylase) |
| AMDHD1 | -1.181435156 | Amidohydrolase domain containing 1 |
| BCKDHB | -1.189409762 | Branched chain keto acid dehydrogenase E1, beta polypeptide |
| ENOPH1 | -1.191315211 | Enolase-phosphatase 1 |
| PAH | -1.194299339 | Phenylalanine hydroxylase |
| HPD | -1.195277616 | 4-hydroxyphenylpyruvate dioxygenase |
| NAGS | -1.201942204 | N-acetylglutamate synthase |
| PDHA2 | -1.218709713 | Pyruvate dehydrogenase (lipoamide) alpha 2 |
| GATM | -1.219696802 | Glycine amidinotransferase (L-arginine:glycine amidinotransferase) |
| DLST | -1.226882616 | Dihydrolipoamide S-succinyltransferase (E2 component of 2-oxo-glutarate complex) |
| MUT | -1.261085255 | Methylmalonyl CoA mutase |
| WARS | -1.261409383 | Tryptophanyl-tRNA synthetase |
| TMLHE | -1.269324034 | Trimethyllysine hydroxylase, epsilon |
| NIT2 | -1.273289919 | Nitrilase family, member 2 |
| ACAT2 | -1.273820581 | Acetyl-CoA acetyltransferase 2 |
| HSD17B10 | -1.279305839 | Hydroxysteroid (17-beta) dehydrogenase 10 |
| HNMT | -1.282112289 | Histamine N-methyltransferase |
| ALAS1 | -1.289133955 | Aminolevulinate, delta-, synthase 1 |
| DBT | -1.291136192 | Dihydrolipoamide branched chain transacylase E2 |
| SRM | -1.307465446 | Spermidine synthase |
| DBT | -1.318813089 | Dihydrolipoamide branched chain transacylase E2 |
| IARS | -1.319263173 | Isoleucyl-tRNA synthetase |
| KYNU | -1.340216646 | Kynureninase |
| ABP1 | -1.344660531 | Amiloride binding protein 1 (amine oxidase (copper-containing)) |
| AADAT | -1.351259452 | Aminoadipate aminotransferase |
| AADAT | -1.374297424 | Aminoadipate aminotransferase |
| ASL | -1.375442968 | Argininosuccinate lyase |
| OGDH | -1.379222693 | Oxoglutarate (alpha-ketoglutarate) dehydrogenase (lipoamide) |
| BBOX1 | -1.381943610 | Butyrobetaine (gamma), 2-oxoglutarate dioxygenase (gamma-butyrobetaine hydroxylase) 1 |
| SARDH | -1.383102642 | Sarcosine dehydrogenase |
| HADH | -1.384212439 | Hydroxyacyl-CoA dehydrogenase |
| SAT1 | -1.391548633 | Spermidine/spermine N1-acetyltransferase 1 |
| AGXT | -1.395042156 | Alanine-glyoxylate aminotransferase |
| INMT | -1.401546508 | Indolethylamine N-methyltransferase |
| GAD2 | -1.434035867 | Glutamate decarboxylase 2 (pancreatic islets and brain, 65kDa) |
| ACADM | -1.455554437 | Acyl-CoA dehydrogenase, C-4 to C-12 straight chain |
| HAAO | -1.603799351 | 3-hydroxyanthranilate 3,4-dioxygenase |
| BHMT | -1.604770335 | Betaine--homocysteine S-methyltransferase |
| ALDH6A1 | -1.672266552 | Aldehyde dehydrogenase 6 family, member A1 |
| CNDP1 | -1.689122836 | Carnosine dipeptidase 1 (metallopeptidase M20 family) |
| GCAT | -1.751840912 | Glycine C-acetyltransferase |
| ECHS1 | -1.766689657 | Enoyl CoA hydratase, short chain, 1, mitochondrial |
| DMGDH | -1.806300429 | Dimethylglycine dehydrogenase |
| PDHB | -1.891747215 | Pyruvate dehydrogenase (lipoamide) beta |
| AOX1 | -1.90376793 | Aldehyde oxidase 1 |
| ADH5 | -2.012444992 | Alcohol dehydrogenase 5 (class III), chi polypeptide |
| ADI1 | -2.077867678 | Acireductone dioxygenase 1 |
| FAH | -2.103061423 | Fumarylacetoacetate hydrolase (fumarylacetoacetase) |
| DAO | -2.174519227 | D-amino-acid oxidase |
| ALDH2 | -2.213792245 | Aldehyde dehydrogenase 2 family (mitochondrial) |
| HADHB | -2.285902714 | Hydroxyacyl-CoA dehydrogenase/3-ketoacyl-CoA thiolase/enoyl-CoA hydratase (trifunctional protein), beta subunit |
| ABAT | -2.316387354 | 4-aminobutyrate aminotransferase |
| SDS | -2.322274481 | Serine dehydratase |
| MTAP | -2.324377744 | Methylthioadenosine phosphorylase |
| IVD | -2.507922223 | Isovaleryl-CoA dehydrogenase |
| DDC | -2.657461524 | Dopa decarboxylase (aromatic L-amino acid decarboxylase) |
| AANAT | -2.762216567 | Aralkylamine N-acetyltransferase |
| ASPA | -2.80059411 | Aspartoacylase |
| PRODH2 | -2.839764756 | Proline dehydrogenase (oxidase) 2 |
| ACMSD | -2.861978719 | Aminocarboxymuconate semialdehyde decarboxylase |
| HMGCL | -3.03586148 | 3-hydroxymethyl-3-methylglutaryl-CoA lyase |
| CDO1 | -3.165272881 | Cysteine dioxygenase, type I |
| MAT1A | -3.222019204 | Methionine adenosyltransferase I, alpha |
| SDS | -3.250511253 | Serine dehydratase |
| OGDHL | -3.289492244 | Oxoglutarate dehydrogenase-like |
| BHMT | -3.475558908 | Betaine--homocysteine S-methyltransferase |
| DAO | -3.493744664 | D-amino-acid oxidase |
