# Supplementary material for: SOX12 promotes colorectal cancer cell proliferation and metastasis by regulating asparagine synthesis
Source: Cell Death Dis. 2019 Mar 11;10(3):239. doi: 10.1038/s41419-019-1481-9 (PMC6412063; doi:10.1038/s41419-019-1481-9)
Supplement: Supplementary file 9 — Supplementary Table S5 [file 41419_2019_1481_MOESM9_ESM.doc]

Supplementary Table S5. Correlation between GLS, GOT2, and ASNS expression and clinicopathological characteristics in human CRC tissues

| Clinicopathological variables | | Tumor GLS expression | | P value |  | Tumor GOT2 expression | | p Value |  | Tumor ASNS expression | | P value |
| --- | --- | --- | --- | --- | --- | --- | --- | --- | --- | --- | --- | --- |
|  |  | Negative (n=225) | Positive (n=165) |  |  | Negative (n=238) | Positive (n=152) |  |  | Negative (n=250) | Positive (n=140) |  |
| Age | | 68.22(11.20) | 66.48(11.98) | 0.536 |  | 68.03(10.62) | 66.64(12.87) | 0.25 |  | 67.91(10.79) | 66.74(12.81) | 0.135 |
| Sex | female | 99 | 78 | 0.538 |  | 106 | 71 | 0.678 |  | 109 | 68 | 0.396 |
|  | male | 126 | 87 |  |  | 132 | 81 |  |  | 141 | 72 |  |
| Tumor location | right colon | 88 | 77 | 0.133 |  | 89 | 76 | 0.04 |  | 99 | 66 | 0.092 |
|  | left colon | 101 | 67 |  |  | 112 | 56 |  |  | 110 | 58 |  |
|  | rectum | 36 | 21 |  |  | 37 | 20 |  |  | 41 | 16 |  |
| Tumor size | ＜5cm | 95 | 54 | 0.059 |  | 97 | 52 | 0.202 |  | 106 | 43 | 0.023 |
|  | ≥5cm | 130 | 111 |  |  | 141 | 100 |  |  | 144 | 97 |  |
| Tumor differentiation | well or moderate | 152 | 72 | <0.001 |  | 161 | 63 | <0.001 |  | 167 | 57 | <0.001 |
|  | poor | 73 | 93 |  |  | 77 | 89 |  |  | 83 | 83 |  |
| Tumor invasion | T1 | 13 | 3 | 0.056 |  | 14 | 2 | 0.007 |  | 16 | 0 | 0.001 |
|  | T2 | 14 | 10 |  |  | 17 | 7 |  |  | 19 | 5 |  |
|  | T3 | 158 | 115 |  |  | 166 | 107 |  |  | 175 | 98 |  |
|  | T4 | 40 | 37 |  |  | 41 | 36 |  |  | 40 | 37 |  |
| Lymph node metastasis | absent | 172 | 54 | <0.001 |  | 185 | 41 | <0.001 |  | 188 | 38 | <0.001 |
|  | present | 53 | 111 |  |  | 53 | 111 |  |  | 62 | 102 |  |
| Distant metastasis | absent | 200 | 118 | <0.001 |  | 223 | 95 | <0.001 |  | 230 | 88 | <0.001 |
|  | present | 25 | 47 |  |  | 15 | 57 |  |  | 20 | 52 |  |
| AJCC stage | Stage I | 16 | 4 | <0.001 |  | 17 | 3 | <0.001 |  | 20 | 0 | <0.001 |
|  | Stage II | 151 | 48 |  |  | 164 | 35 |  |  | 164 | 35 |  |
|  | Stage III | 34 | 67 |  |  | 43 | 58 |  |  | 46 | 55 |  |
|  | Stage IV | 24 | 46 |  |  | 14 | 56 |  |  | 20 | 50 |  |
