# Supplementary material for: SOX12 promotes colorectal cancer cell proliferation and metastasis by regulating asparagine synthesis
Source: Cell Death Dis. 2019 Mar 11;10(3):239. doi: 10.1038/s41419-019-1481-9 (PMC6412063; doi:10.1038/s41419-019-1481-9)
Supplement: Supplementary file 10 — Supplementary Table S6 [file 41419_2019_1481_MOESM10_ESM.docx]

Supplementary Table S6. Correlation between HIF-1α expression and clinicopathological characteristics in human CRC tissues

| Clinicopathological variables | | Tumor HIF-1α expression | | P value |
| --- | --- | --- | --- | --- |
|  |  | Negative (n=215) | Positive (n=175) |  |
| Age | | 68.58(11.15) | 66.15(11.92) | 0.315 |
| Sex | female | 99 | 78 | 0.838 |
|  | male | 116 | 97 |  |
| Tumor location | right colon | 84 | 81 | 0.127 |
|  | left colon | 96 | 72 |  |
|  | rectum | 35 | 22 |  |
| Tumor size | ＜5cm | 81 | 68 | 0.835 |
|  | ≥5cm | 134 | 107 |  |
| Tumor differentiation | well or moderate | 148 | 76 | <0.001 |
|  | poor | 67 | 99 |  |
| Tumor invasion | T1 | 15 | 1 | <0.001 |
|  | T2 | 15 | 9 |  |
|  | T3 | 156 | 117 |  |
|  | T4 | 29 | 48 |  |
| Lymph node metastasis | absent | 174 | 52 | <0.001 |
|  | present | 41 | 123 |  |
| Distant metastasis | absent | 201 | 117 | <0.001 |
|  | present | 14 | 58 |  |
| AJCC stage | Stage I | 18 | 2 | <0.001 |
|  | Stage II | 152 | 47 |  |
|  | Stage III | 31 | 70 |  |
|  | Stage IV | 14 | 56 |  |
