# Supplementary material for: SOX12 promotes colorectal cancer cell proliferation and metastasis by regulating asparagine synthesis
Source: Cell Death Dis. 2019 Mar 11;10(3):239. doi: 10.1038/s41419-019-1481-9 (PMC6412063; doi:10.1038/s41419-019-1481-9)
Supplement: Supplementary file 11 — Supplementary Table S7 [file 41419_2019_1481_MOESM11_ESM.docx]

Supplementary Table S7. Primer sequences used in the study

| Primer name | Primer sequences | Enzyme |  |
| --- | --- | --- | --- |
| Primers for real-time PCR: |  |  |  |
| SOX12 sense: | 5'-CGCGATGGTGCAGCAGCG-3' |  |  |
| SOX12 antisense: | 5'-GCCACTGGTCCATGATCTTC-3' |  |  |
| GLS sense: | 5’-TGCATTCCTGTGGCATGTAT-3’ |  |  |
| GLS antisense: | 5’-TTGCCCATCTTATCCAGAGG-3’ |  |  |
| GOT2 sense: | 5’-GTTTGCCTCTGCCAATCATATG-3’ |  |  |
| GOT2 antisense: | 5’-GAGGGTTGGAATACATGGGAC-3’ |  |  |
| ASNS sense: | 5’-TGCTTACGCCCAGATTTTCT-3’ |  |  |
| ASNS antisense: | 5’-AAAACGGAATGCATCTGGAC-3’ |  |  |
| GAPDH sense: | 5’-GCACCGTCAAGGCTGAGAAC-3’ |  |  |
| GAPDH antisense: | 5’-TGGTGAAGACGCCAGTGGA-3’ |  |  |
| Primers for GLS promoter construct: | |  |  |
| (-2046/+36)GLS sense: | 5’-TATAGGTACCAACTCTAGAAACTAGAAACT-3’ | KpnI |  |
| (-1391/+36)GLS sense: | 5’-TATAGGTACCTTCTAGTTTCATAAAAATCA-3’ | KpnI |  |
| (-550/+36)GLS sense: | 5’-TATAGGTACCTTTCAAGCAAATGTTACATT-3’ | KpnI |  |
| Antisense: | 5’-ATATAAGCTTTTAATTGTTTTAAGAAAATT-3’ | HindIII |  |
| Primers for GLS promoter site-directed mutagenesis: | |  |  |
| binding site 2 mutation sense: | 5’-AAATATCTAAAcagcTTTTCATAAGA-3’ |  |  |
| binding site 2 mutation antisense: | 5’-TCTTATGAAAAgctgTTTAGATATTT-3’ | |  |
| binding site 1 mutation sense: | 5’-TTTAAACCTAAgctgTTCAGGAAATA-3’ | |  |
| binding site 1 mutation antisense: | 5’-TATTTCCTGAAcagcTTAGGTTTAAA-3’ |  |  |
| Primers used for ChIP in the GLS promoter: | |  |  |
| distant region sense: | 5’-ATTTTATTATGATCTTGAAA-3’ |  |  |
| distant region antisense: | 5’-AATTGGAATTTGTGCACTTT-3’ |  |  |
| binding site 2 sense: | 5’-AACTCTAGAAACTAGAAACT-3’ |  |  |
| binding site 2 antisense: | 5’-ATTAGTTATCAATTTTAACT-3’ |  |  |
| binding site 1 sense: | 5’-AAAACATCTTATTAAACTCT-3’ |  |  |
| binding site 1 antisense: | 5’-TAAGTTATTTCAGCATCACTT-3’ |  |  |
| Primers for GOT2 promoter construct: | |  |  |
| (-3786/+102)GOT2 sense: | 5’-TATAGGTACCAAATTAGCATATATACATAT-3’ | KpnI |  |
| (-3009/+102)GOT2 sense: | 5’-TATAGGTACCACATCTATTGAGTTCATTCA-3’ | KpnI |  |
| (-2868/+102)GOT2 sense: | 5’-TATAGGTACCTTAAAAATGTGTGAGAAGAT-3’ | KpnI |  |
| (-940/+102)GOT2 sense: | 5’-TATAGGTACCTTTAAGAGACGAGTATCAGA-3’ | KpnI |  |
| Antisense: | 5’-ATATAAGCTTTTATGATCAGCTACACGATT-3’ | HindIII |  |
| Primers for GOT2 promoter site-directed mutagenesis: | |  |  |
| binding site 3 mutation sense: | 5’-AATGGTGACAAcgacTAATCTGATCT-3’ |  |  |
| binding site 3 mutation antisense: | 5’-AGATCAGATTAgtcgTTGTCACCATT-3’ |  |  |
| binding site 2 mutation sense: | 5’-TGCTTTTATAAgacgTGCTTTACTGA-3’ |  |  |
| binding site 2 mutation antisense: | 5’-TCAGTAAAGCAcgtcTTATAAAAGCA-3’ | |  |
| binding site 1 mutation sense: | 5’-TGGACATGTAAcgacTTTGGGAATAG-3’ | |  |
| binding site 1 mutation antisense: | 5’-CTATTCCCAAAgtcgTTACATGTCCA-3’ |  |  |
| Primers used for ChIP in the GOT2 promoter: | |  |  |
| distant region sense: | 5’-TCTGCATCTAATCACCAAAT-3’ |  |  |
| distant region antisense: | 5’-AATCCAGGCAAGAAATTCTG-3’ |  |  |
| binding site 3 sense: | 5’-AAATTAGCATATATACATAT-3’ |  |  |
| binding site 3 antisense: | 5’-TACAAAAATTGCCAGATGT-3’ |  |  |
| binding site 1,2 sense: | 5’-ACATCTATTGAGTTCATTCA-3’ |  |  |
| binding site 1,2 antisense: | 5’-TGAATGAACTCAATAGATGT-3’ |  |  |
| Primers for ASNS promoter construct: | |  |  |
| (-1991/+67)ASNS sense: | 5'-TATAGGTACCATAAATTGTAAAGATTTCAT-3' | KpnI |  |
| (-1172/+67)ASNS sense: | 5’-TATAGGTACCTTTACACAGATTATGACTCA-3’ | KpnI |  |
| (-971/+67)ASNS sense: | 5’-TATAGGTACCTTAGAACAAGTCATCTCTCA-3’ | KpnI |  |
| (-641/+67)ASNS sense: | 5’-TATAGGTACCAAGTTGACAAAATCCTTTTT-3’ | KpnI |  |
| (-271/+67)ASNS sense: | 5’-TATAGGTACCTTATTAATAGATCTTATAAG-3’ | KpnI |  |
| Antisense: | 5’-ATATAAGCTTTTTACTGTGATACAGCAAAA-3’ | HindIII |  |
| Primers for ASNS promoter site-directed mutagenesis: | |  |  |
| binding site 4 mutation sense: | 5’-GATAACAGCAAcgagTCAGGGAATAA-3’ | |  |
| binding site 4 mutation antisense: | 5’-TTATTCCCTGActcgTTGCTGTTATC-3’ | |  |
| binding site 3 mutation sense: | 5’-ACTTTCCAGCAgagcTGAACCTTAGA-3’ | |  |
| binding site 3 mutation antisense: | 5’-TCTAAGGTTCAgctcTGCTGGAAAGT-3’ | |  |
| binding site 2 mutation sense: | 5’-GGGATTTTAAAcgagTGACGTTTTTG-3’ | |  |
| binding site 2 mutation antisense: | 5’-CAAAAACGTCActcgTTTAAAATCCC-3’ |  |  |
| binding site 1 mutation sense: | 5’-TTTAGATTTTAcgagTTTAGAGATGG-3’ |  |  |
| binding site 1 mutation antisense: | 5’-CCATCTCTAAActcgTAAAATCTAAA-3’ | |  |
| Primers used for ChIP in the ASNS promoter: | |  |  |
| distant region sense: | 5’-TCTACTAAGTACAAAAATTA-3’ |  |  |
| distant region antisense: | 5’-ATTTGTTTGTTTTTGAGACA-3’ |  |  |
| binding site 4 sense: | 5’-TTAAGTACACAAATTATACA-3’ |  |  |
| binding site 4 antisense: | 5’-CATTTGCTTTTGAAAGAAGA-3’ |  |  |
| binding site 2,3 sense: | 5’-ATACAGCAAAGTCTGTTTGA-3’ |  |  |
| binding site 2,3 antisense: | 5’-ATAGTAAAATTATATGTGCA-3’ |  |  |
| binding site 1 sense: | 5’-AAGTTGACAAAATCCTTTTT-3’ |  |  |
| binding site 1 antisense: | 5’-CTGAGGCAGAAGGATAGTTT-3’ |  |  |
| Primers for SOX12 promoter construct: | |  |  |
| (-1526/+28)SOX12 sense: | 5’-TATAGGTACCTACATGCATTATCTCATTTA-3’ | KpnI |  |
| (-1357/+28)SOX12 sense: | 5’-ATATGGTACCTTCCAGATCTCAGCTTCTGA-3’ | KpnI |  |
| (-809/+28)SOX12 sense: | 5’-ATATGGTACCCTTCTCCAGGCTTGGGTGGG-3’ | KpnI |  |
| (-708/+28)SOX12 sense: | 5’-ATATGGTACCTCGCCTGCGGTTTGTGAAAA-3’ | KpnI |  |
| (-428/+28)SOX12 sense: | 5’-ATATGGTACCATTAAATATACATATATATG-3’ | KpnI |  |
| antisense: | 5’-ATATAAGCTTTTTCTCTAGAAACAGACAAT-3’ | HindIII |  |
| Primers for SOX12 promoter site-directed mutagenesis: | |  |  |
| HIF-1α binding site: |  |  |  |
| binding site 5 mutation sense: | 5’-TAAAGGTTTTAtcatgcGGCTTTTCCAG-3’ |  |  |
| binding site 5 mutation antisense: | 5’-CTGGAAAAGCCgcatgaTAAAACCTTTA-3’ |  |  |
| binding site 4 mutation sense: | 5’-CAGGGGCGGCAtgatatCAGTGGTGCCC-3’ |  |  |
| binding site 4 mutation antisense: | 5’-GGGCACCACTGatatcaTGCCGCCCCTG-3’ |  |  |
| binding site 3 mutation sense: | 5’-GAACTGGCTCAagatcaGTGAGCCGGGA-3’ |  |  |
| binding site 3 mutation antisense: | 5’-TCCCGGCTCACtgatctTGAGCCAGTTC-3’ |  |  |
| binding site 2 mutation sense: | 5’-AATGGCGCACCagatagTCCTCGAATGT-3’ |  |  |
| binding site 2 mutation antisense: | 5’-ACATTCGAGGActatctGGTGCGCCATT-3’ |  |  |
| binding site 1 mutation sense: | 5’-GAATCCTAGTCgatagaTCCTCGGTGCTT-3’ |  |  |
| binding site 1 mutation antisense: | 5’-AAGCACCGAGGAtctatcGACTAGGATTC-3’ |  |  |
| Primers used for ChIP in the SOX12 promoter: | |  |  |
| distant region sense: | 5’-TGAGAATAAAGAGTGAAATC-3’ |  |  |
| distant region antisense: | 5’-AGCTTTCTTGGAATTATCAA-3’ |  |  |
| binding site 5 sense: | 5’-TACATGCATTATCTCATTTA-3’ |  |  |
| binding site 5 antisense: | 5’-TCAGAAGCTGAGATCTGGAA-3’ |  |  |
| binding site 3,4 sense: | 5’-ACCTTAAAGACCCTAAT-3’ |  |  |
| binding site 3,4 antisense: | 5’-CCCACCCAAGCCTGGAGAAG-3’ |  |  |
| binding site 2 sense: | 5’-CTTCTCCAGGCTTGGGTGGG-3’ |  |  |
| binding site 2 antisense: | 5’-TTTTCACAAACCGCAGGCGA-3’ |  |  |
| binding site 1 sense: | 5’-TCGCCTGCGGTTTGTGAAAA-3’ |  |  |
| binding site 1 antisense: | 5’-CATATATATGTATATTTAAT-3’ |  |  |
