# Supplementary material for: SOX12 promotes colorectal cancer cell proliferation and metastasis by regulating asparagine synthesis
Source: Cell Death Dis. 2019 Mar 11;10(3):239. doi: 10.1038/s41419-019-1481-9 (PMC6412063; doi:10.1038/s41419-019-1481-9)
Supplement: Supplementary file 12 — Supplementary Table S8 [file 41419_2019_1481_MOESM12_ESM.doc]

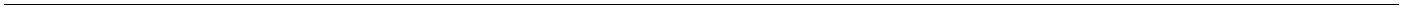

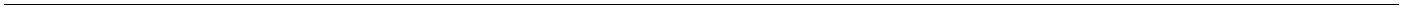
Supplementary Table S8. Knockdown shRNA sequences used in this study

**TRC number Sequence**

**SOX12**

TRCN0000019020 CCGGCATGGCGGATTACCCGGACTACTCGAGTAGTCCGGGTAATCCGCCATGTTTTT

TRCN0000019023 CCGGGCTGGGCTTTCTGTCCAGGCTCTCGAGAGCCTGGACAGAAAGCCCAGCTTTTT

**GLS**

TRCN0000051135 CCGGGCACAGACATGGTTGGTATATCTCGAGATATACCAACCATGTCTGTGCTTTTTG

TRCN0000051136 CCGGGCCCTGAAGCAGTTCGAAATACTCGAGTATTTCGAACTGCTTCAGGGCTTTTTG

**GOT2**

TRCN0000034827 CCGGGCTACAAGGTTATCGGTATTACTCGAGTAATACCGATAACCTTGTAGCTTTTTG

TRCN0000034828 CCGGGCCTTTAAGAGGGACACCAATCTCGAGATTGGTGTCCCTCTTAAAGGCTTTTTG

**ASNS**

TRCN0000290103 CCGGCGTCAAGTCTTTGAACGCCATCTCGAGATGGCGTTCAAAGACTTGACGTTTTTG


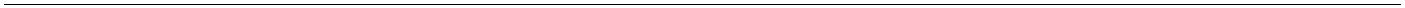
TRCN0000290105 CCGGCGAGTGAAGAAATATCCGTATCTCGAGATACGGATATTTCTTCACTCGTTTTTG
