# Supplementary material for: SOX12 promotes colorectal cancer cell proliferation and metastasis by regulating asparagine synthesis
Source: Cell Death Dis. 2019 Mar 11;10(3):239. doi: 10.1038/s41419-019-1481-9 (PMC6412063; doi:10.1038/s41419-019-1481-9)
Supplement: Supplementary file 13 — Supplemental Figure legends [file 41419_2019_1481_MOESM13_ESM.docx]

**Supplementary figure legends**

**Supplementary Figure S1. Expression of SOX12 in The Cancer Genome Atlas (TCGA) datasets.** (A) Representative data extracted from The Cancer Genome Atlas (TCGA) dataset showing the relative mRNA expression of SOX12 in multiple cancer versus normal tissues. Box-and-whisker plots indicating the median (horizontal line), interquartile range (box) and min to max (whiskers) of the data. COAD, Colon Adenocarcinoa: LIHC, Liver hepatocellular carcinoma; STAD, Stomach Adenocarcinoma; ESCA, Esophageal Carcinoma; KIRC, Kidney renal clear cell carcinoma; BRCA, Breast invasive carcinoma; Lung adenocarcinoma, LUAD; UCEC, Uterine Corpus Endometrial Carcinoma.

**Supplementary Figure S2. The mRNA (A) and protein (B) expression of SOX12 in colorectal cancer cell lines.**

**Supplementary Figure S3. SOX12 promotes CRC cell proliferation, migration and invasion *in vitro.***

(A) Western blots showing the effects of lentiviral infection of the indicated human CRC cells. (B) The effects of SOX12 on CRC cell proliferation were measured using a CCK-8 assay. (C) Effects of SOX12 on human CRC cell colony formation. (D) Transwell assays using the indicated cells. *P<0.05 compared with the control. The data are presented as the mean ±s.d.

**Supplementary Figure S4. (A) SOX12 expression in the indicated cells as determined by western blot analysis. (B)Relative cell migration in the indicated cells as determined by transwell assays.**
